# Smartphone-based detection of subtle memory decline in prodromal Alzheimer’s disease

**DOI:** 10.1038/s41746-026-02731-1

**Published:** 2026-06-10

**Authors:** Sarah E. Polk, Lindsay R. Clark, Kristin Basche, Luca Kleineidam, Wenzel Glanz, Michaela Butryn, Robert Perneczky, Katharina Buerger, Klaus Fliessbach, Christoph Laske, Annika Spottke, Anja Schneider, Jens Wiltfang, Stefan Teipel, Claudia Bartels, Ayda Rostamzadeh, Daniel Janowitz, Boris-Stephan Rauchmann, Ingo Kilimann, Sebastian Sodenkamp, Marie Coenjaerts, Frederic Brosseron, Michael Wagner, Ingo Frommann, Melina Stark, Matthias Schmid, Björn H. Schott, Sterling C. Johnson, Frank Jessen, Emrah Düzel, David Berron

**Affiliations:** 1 German Center for Neurodegenerative Diseases (DZNE), Magdeburg, Germany; 2 Division of Geriatrics, Department of Medicine, University of Wisconsin School of Medicine and Public Health, Madison, WI USA; 3 Wisconsin Alzheimer’s Disease Research Center, Madison, WI USA; 4 Geriatric Research, Education and Clinical Center, William S. Middleton Memorial Veterans Hospital, Madison, WI USA; 5 German Center for Neurodegenerative Diseases (DZNE), Bonn, Germany; 6 Department for Cognitive Disorders and Old Age Psychiatry, University Hospital Bonn, Bonn, Germany; 7 German Center for Neurodegenerative Diseases (DZNE), Munich, Germany; 8 Department of Psychiatry and Psychotherapy, University Hospital, LMU Munich, Munich, Germany; 9 Munich Cluster for Systems Neurology (SyNergy), Munich, Germany; 10 Ageing Epidemiology Research Unit (AGE), School of Public Health, Imperial College London, London, UK; 11 Institute for Stroke and Dementia Research (ISD), University Hospital, LMU Munich, Munich, Germany; 12 Department of Old Age Psychiatry and Cognitive Disorders, University Hospital Bonn and University of Bonn, Bonn, Germany; 13 German Center for Neurodegenerative Diseases (DZNE), Tübingen, Germany; 14 Section for Dementia Research, Hertie Institute for Clinical Brain Research and Department of Psychiatry and Psychotherapy, University of Tübingen, Tübingen, Germany; 15 Department of Neurology, University of Bonn, Bonn, Germany; 16 German Center for Neurodegenerative Diseases (DZNE), Goettingen, Germany; 17 Department of Psychiatry and Psychotherapy, University Medical Center Goettingen, University of Goettingen, Goettingen, Germany; 18 Neurosciences and Signaling Group, Institute of Biomedicine (iBiMED), Department of Medical Sciences, University of Aveiro, Aveiro, Portugal; 19 German Center for Neurodegenerative Diseases (DZNE), Rostock, Germany; 20 Department of Psychosomatic Medicine, Rostock University Medical Center, Rostock, Germany; 21 Department of Psychiatry, University of Cologne, Medical Faculty, Cologne, Germany; 22 Sheffield Institute for Translational Neuroscience (SITraN), University of Sheffield, Sheffield, UK; 23 Department of Neuroradiology, University Hospital LMU, Munich, Germany; 24 Institute for Medical Biometry, Informatics and Epidemiology, University Hospital Bonn, Bonn, Germany; 25 Leibniz Institute for Neurobiology, Magdeburg, Germany; 26 Excellence Cluster on Cellular Stress Responses in Aging-Associated Diseases (CECAD), University of Cologne, Cologne, Germany; 27 Institute of Cognitive Neurology and Dementia Research (IKND), Otto-von-Guericke University, Magdeburg, Germany; 28 Center for Behavioral Brain Sciences, Otto-von-Guericke University Magdeburg, Magdeburg, Germany; 29 Clinical Memory Research Unit, Department of Clinical Sciences, Lund University, Lund, Sweden

**Keywords:** Biomarkers, Neurology, Neuroscience

## Abstract

The slow progression of Alzheimer’s disease (AD) poses a challenge for the quantification of early disease-driven cognitive decline. Here, we show that frequently administered remote and unsupervised digital cognitive assessments can detect differences in cognitive decline within 30 weeks in early AD. The sample comprised 202 individuals (52–85 years old) recruited from longitudinal observational studies, who were cognitively unimpaired (CU, *n* = 152) or had a diagnosis of mild cognitive impairment (MCI, *n* = 50). Participants self-administered remote tasks testing memory precision for objects and scenes, associative memory, and familiarity-dependent memory. The MCI group showed greater decline than the CU group in the familiarity-dependent task, while stratifying the MCI group by beta-amyloid (Aβ) status (*n* = 21 Aβ−; *n* = 24 Aβ+) revealed greater change in memory precision for objects and familiarity-dependent memory in the MCI Aβ+ group. A 30-week change in the remote familiarity-dependent task was correlated with a multi-year change in annual in-person neuropsychological assessments. In conclusion, frequent remote cognitive testing is a promising tool to feasibly capture and monitor subtle and short-term cognitive decline.

## Introduction

Memory decline is one of the earliest observed clinical symptoms related to Alzheimer’s disease (AD) pathology^1^, with patient and caregiver reports of progressive memory loss being core diagnostic components of the clinical syndrome. The speed of memory deterioration is also a leading cause of worry for AD patients^2^. Slowing the rate of memory decline is therefore a crucial goal for disease modifying treatments, and the rate of cognitive change is a key outcome measure in clinical trials, especially in earlier disease stages^3–6^. However, despite being a primary target for interventions, it is still not common practice to quantify the rate of memory decline for use in clinical decision making. A primary reason for this is the lack of tools that can reliably measure decline within reasonable time periods appropriate for timely clinical management.

Suitable cognitive tools could be optimized to detect subtle cognitive decline (i.e., short-term decline that cannot be captured by traditional neuropsychological assessments) in AD through two approaches. Firstly, clinician-administered neuropsychological tests are typically performed every 6 to 12 months and show sensitivity to group-level change after 18 to 24 months^7^. Increasing the frequency of measurement would improve the reliability of cognitive tests, thereby improving their sensitivity to change^8^. Secondly, memory function is not monolithically compromised in early AD, but rather those component processes that rely on brain regions in which tau pathology first accumulates and which show early signs of neurodegeneration are those that show the earliest decline^9^. Thus, a tool that targets those processes which are expected to deteriorate earliest may be more sensitive to cognitive decline in early AD.

The advent of remote digital cognitive testing via individuals’ own devices has greatly facilitated the frequent administration of cognitive tests (see refs. ^10,11^ for reviews) in a decentralized manner^12^. In contrast to clinician-administered cognitive tests, it is feasible to administer remote and unsupervised cognitive tests up to multiple times a day in older adults^13–15^. Increasing test frequency theoretically improves the characterization of intra-individual variability, reducing the overall susceptibility to measurement error and improving reliability, which in turn improves the measurement of true cognitive change^8^.

Regarding the specificity of cognitive tests to certain memory processes, many commonly used neuropsychological tests broadly assess memory decline as a general construct. However, given what is known about the spatiotemporal progression of AD pathologies, namely the accumulation of beta-amyloid (Aβ) plaques and tau neurofibrillary tangles^16,17^, it is hypothesized that specific episodic memory processes are affected sequentially in early AD, aligning with the progression of pathology^9,18^. Briefly, the progression of neurofibrillary tangles follows a stereotypical spatiotemporal pattern, accumulating first in the locus coeruleus^19^, followed by the (trans)entorhinal cortex and hippocampus in the medial temporal lobe, and later progressing towards the lateral temporal and parietal cortices^20,21^. The progression of Aβ plaques follows a more diffuse pattern, starting in the neocortex and later affecting other cortical regions, including the entorhinal cortex and hippocampus, subcortical regions, and the brainstem^22–25^.

To implement both of these approaches in the current study, we frequently administered three non-verbal visual memory tasks that recruit brain regions known to be affected by AD pathology. The Mnemonic Discrimination Task for Objects and Scenes (MDT-OS) was designed to tap into pattern separation, or the neural process by which similar stimuli are separated into distinct representations^26–28^; successful pattern separation promotes greater memory precision. This task has been shown to activate a posterior-medial network for scenes, which includes superior occipital and parietal regions, posterior midline regions, and medial temporal regions, including the parahippocampal cortex, as well as an anterior-temporal network for objects, which includes occipital and inferior parietal regions, lateral temporal regions, and the perirhinal cortex^29–31^. The Object-in-Room Recall (ORR) task was designed to recruit pattern completion, or the neural process by which partial memories are completed from previously experienced episodes. This process relies on the hippocampal *cornu ammonis* 3 (CA3)^32,33^. Successful pattern completion supports associative memory, or the ability to bind a stimulus to a certain context and retrieve this stimulus when cued. Finally, the Complex Scene Recognition (CSR) task was designed to measure recognition of previously seen stimuli, reliant on recollection and familiarity. The CSR recruits a larger network of brain regions, including the medial temporal lobe and frontal and parietal regions^34–36^. The encoding of novel stimuli is associated with deactivation of midline parietal and frontal regions (i.e., parts of the default mode network)^37^, while retrieval depends on a network of hippocampal, midline parietal, and frontal regions. Notably, familiarity-dependent recognition memory has been shown to be preserved in individuals with hippocampal injury^38^, while it is impaired in cases with perirhinal and parietal damage but preserved hippocampal integrity^39^.

**Fig. 1:**
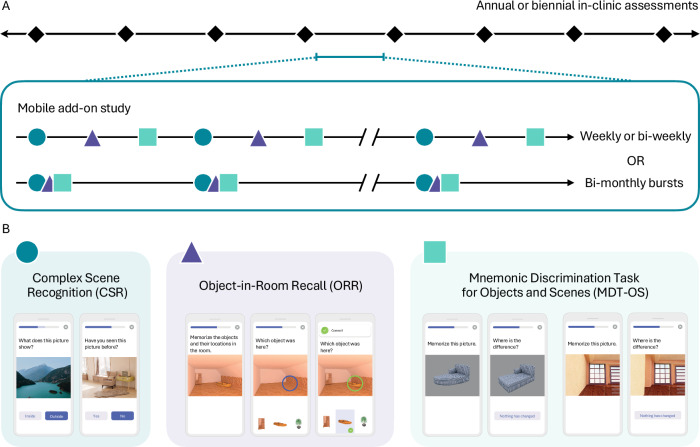
Study design. **A** Participants were recruited from ongoing parent studies with annual or biennial in-clinic assessments (diamonds) to participate in the mobile add-on study in one of two possible designs: a continuous design with weekly or biweekly remote assessments or a burst design with four-day bursts every second month. **B** The tasks included a task for familiarity-dependent memory (Complex Scene Recognition or CSR; circles), a task for associative memory (Object-in-Room Recall or ORR; triangles), and a task for memory precision of objects and scenes (Mnemonic Discrimination Task for Objects and Scenes or MDT-OS; squares).

For the current analyses, these three remote and unsupervised tasks capturing memory precision, associative memory, and familiarity-dependent memory were administered to a sample including cognitively unimpaired (CU) individuals and patients with mild cognitive impairment (MCI) weekly or every other week for at least 30 weeks, up to a year (see Fig. 1). Task performance was analyzed to investigate subtle disease-driven changes in cognition.

We first examined the feasibility of high-frequency remote assessments in the current sample, focusing on adherence and compliance to the assigned schedule.

Next, we were interested in whether remote and unsupervised memory assessments are sensitive to differences in subtle cognitive change between CU and MCI groups. We first confirmed that the current sample of MCI patients showed overall cognitive decline compared to CU individuals as measured with gold standard in-clinic neuropsychological assessments; we tested for differences in change in cognitive performance over eight years on a latent measure similar to the Preclinical Alzheimer’s cognitive composite (PACC), which is sensitive to longer-term decline (18 to 24 months) even in cognitively healthy individuals with biomarker evidence for AD^7,40^. After confirming that the current group with MCI showed greater long-term cognitive decline as expected, we then tested whether this same group would show greater short-term change on remote digital cognitive assessments. We hypothesized that CU participants would show relatively stable trajectories when controlling for procedural practice effects, while MCI patients would show more negative change in performance over time^41^. Additionally, we stratified the MCI group according to Aβ status, hypothesizing that those patients with MCI due to AD (MCI Aβ+) would decline more steeply than those with other-cause MCI (MCI Aβ−).

During the prodromal stage of AD (MCI due to AD), the medial temporal lobe already shows tau PET uptake, while the neocortex starts to show moderate tau PET uptake^42^. We therefore hypothesized that performance on the ORR and MDT-OS would already be impaired in the current MCI sample (i.e., worse than the CU group at baseline), especially in those with amyloid positivity. Performance on the CSR (familiarity-dependent memory) may be relatively spared, even as hippocampal integrity is compromised, due to its reliance on additional processes such as familiarity and therefore additional extra hippocampal brain regions^43^. We hypothesized that CSR performance would begin to decline during prodromal AD as the neocortex, including posterior parietal areas, begins to be affected.

Finally, we examined the associations between change in remote task performance and established in-person neuropsychological measures. We also investigated whether in-person tests were sensitive to short-term cognitive changes in a subsample of participants with two in-person assessments within one year.

## Results

### Participants

Volunteers aged 50 years or older were recruited for an unsupervised and remote study (mobile add-on study) through three longitudinal observational cohort studies (DZNE: Longitudinal Cognitive Impairment and Dementia Study [DELCODE]^44^, Wisconsin Registry for Alzheimer’s Prevention [WRAP]^45^, Wisconsin Alzheimer’s Disease Research Center [ADRC]), as well as from the local memory clinic. We included 202 eligible participants from the remote mobile add-on study in the current analysis, comprising 152 CU participants and 50 participants with an MCI diagnosis. All CU participants included had healthy Aβ levels (CU Aβ−); 21 of the individuals with MCI were also Aβ−, while 24 were Aβ+. Sample characteristics and summaries of key measures at baseline can be found in Table 1.

**Table 1:** Participant demographics, information about remote testing, and baseline scores on key measures

	All	CU	MCI	Group difference
Participants	202	152 (75%)	50 (25%)	
Aβ status	5 missing	0 missing	5 missing	
Aβ−	173 (86%)	152 (100%)	21 (42%)	
Aβ+	24 (12%)	—	24 (48%)	
Study cohort				
DELCODE	77 (38%)	60 (39%)	17 (34%)	*χ*^*2*^_2, 202_ = 96.35*
Memory clinic	34 (17%)	4 (3%)	30 (60%)	
Wisconsin	91 (45%)	88 (58%)	3 (6%)	
Age at start of mobile add-on study (years)	69.9 ± 6.8	69.2 ± 6.6	72.1 ± 7.1	*F*_1, 200_ = 7.10*
Sex (female)	118 (58%)	98 (64%)	20 (40%)	*χ*^*2*^_1, 202_ = 8.30*
Education (years)	15.4 ± 2.7	15.9 ± 2.5	13.8 ± 2.6	*F*_1, 200_ = 25.98*
MMSE	28.5 ± 2.6	29.5 ± 0.7	25.8 ± 3.8	*F*_1, 183_ = 122.46*
CDR sum of boxes	0.2 ± 0.5	0.1 ± 0.3	0.7 ± 0.9	*F*_1, 147_ = 29.72*
Device type	29 missing	24 missing	5 missing	
Smartphone	129 (64%)	96 (63%)	33 (66%)	*χ*^*2*^_1, 193_ = 0.00
Tablet	44 (22%)	32 (21%)	12 (24%)	
Screen size (cm)	16.9 ± 5.1	16.9 ± 5.0	16.9 ± 5.4	*F*_1, 171_ = 0.00
Number of remote sessions completed	22.6 ± 10.3	22.5 ± 8.8	22.8 ± 14.0	*F*_1, 197_ = 0.03
Time spent in mobile add-on study (weeks)	39.6 ± 20.6	42.7 ± 19.2	30.1 ± 21.9	*F*_1, 197_ = 15.04*
Baseline performance				
lPACC	−0.64 ± 1.32	−0.06 ± 0.73	−2.36 ± 1.16	*F*_1, 194_ = 314.50*
Memory precision for objects (MDT-O)	0.51 ± 0.20	0.54 ± 0.18	0.39 ± 0.21	*F*_1, 171_ = 22.27*
Memory precision for scenes (MDT-S)	0.42 ± 0.21	0.48 ± 0.18	0.24 ± 0.19	*F*_1, 171_ = 58.29*
Associative memory (ORR)	19.7 ± 2.7	20.4 ± 2.2	16.1 ± 2.3	*F*_1, 152_ = 83.07*
Familiarity-dependent memory (CSR)	0.53 ± 0.20	0.58 ± 0.18	0.35 ± 0.18	*F*_1, 170_ = 57.62*

*N*s and percentages of all participants in the (sub-) sample or means and standard deviations are reported. MMSE, CDR sum of boxes, and lPACC from the in-person assessment chronologically closest to the start of the mobile add-on study are reported. Results from an ANCOVA controlling for age, sex, years of education, and study cohort are reported for the MMSE, CDR sum of boxes, and lPACC; concentration, distraction, time of day, and time to retrieval (for ORR and CSR) were additionally included for remote task scores. Note that the CDR sum of boxes was not available for memory clinic participants.

*CU* cognitively unimpaired, *MCI* mild cognitive impairment, *MMSE* Mini-Mental State Examination, *CDR* Clinical Dementia Rating, *lPACC* latent Preclinical Alzheimer’s Cognitive Composite, *MDT-O/-S* Mnemonic Discrimination Task for Objects/Scenes (corrected hit rate), *ORR* Object-in-Room Recall (score out of 25), *CSR* Complex Scene Recognition Task (corrected hit rate).

*p < 0.050, indicating a significant difference across groups.

### Adherence and compliance with the remote and unsupervised tests are acceptable

**Fig. 2:**
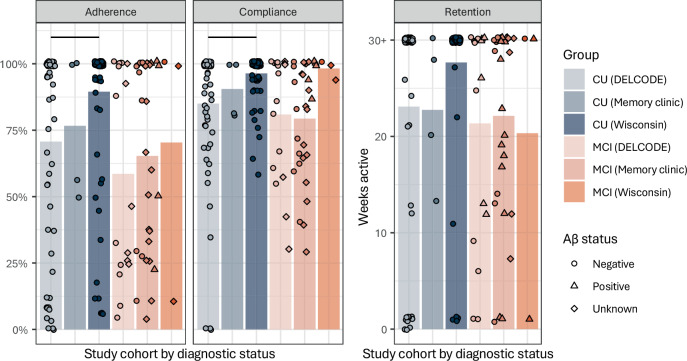
Adherence and compliance with the mobile add-on study indicated good feasibility. By week 30, 147 participants (73%) were still actively engaged in the mobile add-on study, as shown by participant retention. CU cognitively unimpaired, MCI mild cognitive impairment. Solid bars indicate pairwise group differences in estimated marginal means at *p* < 0.050.

To ensure that frequent administration of remote and unsupervised digital cognitive assessments was feasible in the current sample, we examined adherence and compliance to the study schedule, stratifying by cognitive status and study cohort (WRAP and Wisconsin ADRC participants combined into the Wisconsin cohort as their protocols were identical; see Fig. 2). Here, we define adherence as the percentage of tasks completed, while compliance was calculated as the number of task sessions completed divided by how many tasks an individual could have completed given how long they participated in the mobile add-on study (e.g., if a participant from the memory clinic was in the study for 10 weeks, they should have completed ten tasks). Participant retention is defined as the number of weeks spent active in the study.

Overall, there was an average adherence rate of 77% (SD = 34%) and a compliance rate of 89% (SD = 19%). Linear regression models including the effects of cognitive status, study cohort, and cognitive status-by-study cohort were run for adherence and compliance, accounting for demographics. Significant main effects of study cohort [Wisconsin] were found for adherence, *b* = 0.158, *p* = 0.007, and compliance, *b* = 0.125, *p* < 0.001. *Post hoc* comparisons within each group revealed a significant difference between DELCODE and Wisconsin cohorts within the CU group in both adherence, *b* = −0.158, *p* = 0.019, and compliance, *b* = −0.125, *p* = 0.001.

A supplementary analysis investigating factors increasing the probability of drop-out before week 30 was also carried out. Briefly, both cognitive and Aβ status were unrelated to drop-out likelihood, *p*s > 0.524. Being a part of the Wisconsin cohort, younger age, and female sex were associated with a lower probability of dropping out (see Supplementary Table 1 and accompanying text for details).

### Characterization of long-term cognitive decline with in-person neuropsychological tests

Before addressing our aims regarding performance on the remotely administered cognitive tasks, we first characterized cognitive decline measured with gold standard metrics using a cross-cohort harmonized latent PACC (lPACC). Briefly, in-clinic neuropsychological assessments were conducted annually or biennially. As the tests varied somewhat across study cohorts, a confirmatory factor analysis (CFA) approach was used to create a harmonized lPACC score for each individual at each time point (see Methods for more details). Group differences in change in the lPACC were investigated using linear mixed models (LMMs). We included cognitive status, time, and the cognitive status-by-time interaction term, as well as demographic covariates, in the model. A second model was also run, stratifying the MCI group by Aβ status.

**Fig. 3:**
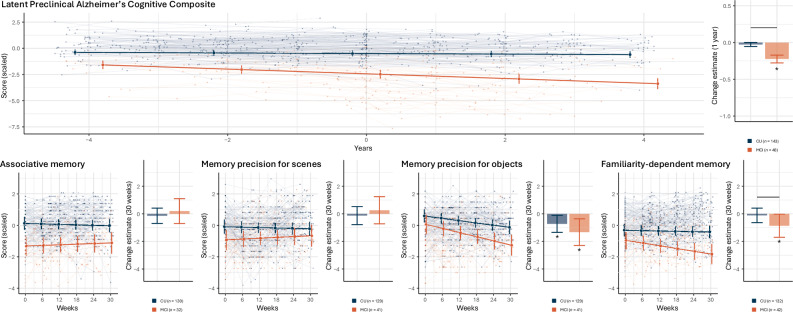
Estimated longitudinal performance trajectories according to cognitive status. Those with a mild cognitive impairment (MCI) diagnosis showed significantly greater decline than the cognitively unimpaired (CU) group on the latent Preclinical Alzheimer’s Cognitive Composite (lPACC) over eight years. Associative memory and memory precision for scenes did not reveal group differences in change, while the MCI group showed significantly greater decline than the CU group in familiarity-dependent memory and memory precision for objects. Error bars represent 95% confidence intervals of the estimated marginal means. Solid bars represent a significant group-by-time interaction effect at *p* < 0.050. * within-group difference significantly different from 0 at *p* < 0.050.

**Fig. 4:**
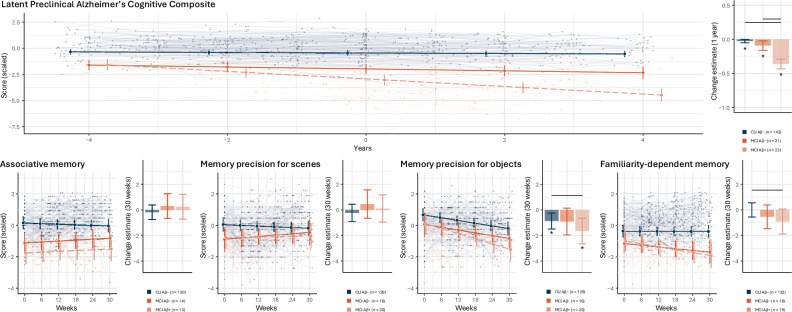
Estimated longitudinal performance trajectories according to cognitive and Aβ status. Those with a mild cognitive impairment (MCI) diagnosis and Aβ positivity showed significantly greater decline than the cognitively unimpaired (CU) group and the MCI Aβ− group on the latent Preclinical Alzheimer’s Cognitive Composite (lPACC) over eight years. Associative memory and memory precision for scenes did not reveal group differences in change, while the MCI Aβ+ group showed significantly greater decline than the CU group in familiarity-dependent memory and memory precision for objects. Error bars represent 95% confidence intervals of the estimated marginal means. Solid bars represent a significant group-by-time interaction effect at *p* < 0.050. * within-group difference significantly different from 0 at *p* < 0.050.

When comparing CU participants and all MCI patients, the MCI group performed worse at baseline, *b* = −1.986, 95% CI [−2.422, −1.554], and showed a significantly more negative slope, *b* = −0.198, 95% CI [−0.258, −0.139] (see Fig. 3). When stratifying the MCI group according to Aβ status, the MCI Aβ− group performed worse than the CU group at the start of the mobile add-on study, *b* = −1.529, 95% CI [−2.002, −1.058], as did the MCI Aβ+ group, *b* = −2.583, 95% CI [−3.101, −2.068] (see Fig. 4). Significant differences in change compared to the CU group were observed for the MCI Aβ+ group, *b* = −0.337, 95% CI [−0.412, −0.263], but not the MCI Aβ− group, *b* = −0.066, 95% CI [−0.140, 0.006]. Pairwise comparisons revealed greater decline in the MCI Aβ+ group versus the MCI Aβ− group, *b* = −0.270, 95% CI [−0.389, −0.151]. See Supplementary Tables 2 and 3 for full results.

### Remote and unsupervised assessments capture differences in short-term decline

Participants completed remote and unsupervised cognitive testing on their own smart devices for at least 30 weeks. The MDT-OS (memory precision) is a lure discrimination task with 32 objects and 32 scene items with one- and two-back phases. The corrected hit rate, that is, the hit rate (percentage of correctly identified repeat items) minus the false alarm rate (percentage of incorrectly identified lure items), was calculated across phases for objects (MDT-O) and scenes (MDT-S) separately. The ORR (associative memory) is a 25-item room-object-location association task with immediate and delayed recall phases. The average score between phases was used as an outcome measure. Finally, the CSR (familiarity-dependent memory) is a delayed recognition task with 60 photographic images depicting scenes. The corrected hit rate was used as an outcome measure. See Methods for more details regarding the tasks.

To capture group differences in performance on the remote tasks, LMMs were run comparing the CU and whole MCI groups, then again stratifying the MCI group by Aβ status. Cognitive status, time, and the cognitive status-by-time interaction term were included in the model, along with demographic and contextual covariates. For the sake of visual clarity, all statistics of interest are reported in Table 2.

**Table 2:** Model estimates of the main effect of group (difference at baseline) on task performance and group-by-time interaction effect (difference in change), and within-group estimated marginal means

		CU vs. all MCI			CU vs. MCI Aβ− and MCI Aβ+				
		Group differences	Within-group EMMs		Group differences		Within-group EMMs		
	Task	CU vs. MCI	CU	MCI	CU vs. MCI Aβ−	CU vs. MCI Aβ+	CU	MCI Aβ−	MCI Aβ+
Baseline	Associative memory	**−1.443** **[−1.913, −0.973]**	0.035 [−0.339, 0.409]	**−1.408** **[−1.923, −0.894]**	**−1.281** **[−1.808, −0.752]**	**−1.907** **[−2.507, −1.310]**	0.041 [−0.331, 0.414]	**−1.239** **[−1.823, −0.656]**	**−1.866** **[−2.498, −1.233]**
	Memory precision for scenes	**−0.844** **[−1.337, −0.354]**	−0.248 [−0.667, 0.171]	**−1.092** **[−1.656, −0.528]**	**−0.950** **[−1.533, −0.368]**	**−0.847** **[−1.434, −0.262]**	−0.173 [−0.591, 0.245]	**−1.123** **[−1.768, −0.477]**	**−1.020** **[−1.673, −0.367]**
	Memory precision for objects	**−0.537** **[−1.015, −0.059]**	0.153 [−0.232, 0.537]	−0.384 [−0.924, 0.156]	**−0.600** **[−1.151, −0.047]**	**−0.562** **[−1.118, −0.008]**	0.195 [−0.188, 0.578]	−0.405 [−1.012, 0.202]	−0.367 [−0.982, 0.247]
	Familiarity-dependent memory	**−0.662** **[−1.083, −0.240]**	−0.192 [−0.540, 0.156]	**−0.854** **[−1.327, −0.382]**	**−0.790** **[−1.259, −0.322]**	**−0.643** **[−1.158, −0.127]**	**−0.365** **[−0.721, −0.010]**	**−1.156** **[−1.695, −0.616]**	**−1.009** **[−1.572, −0.445]**
Change	Associative memory	0.587 [−0.382, 1.553]	−0.240 [−1.203, 0.722]	0.347 [−1.217, 1.910]	0.843 [−0.322, 2.000]	0.752 [−0.377, 1.889]	−0.318 [−1.301, 0.664]	0.525 [−1.164, 2.213]	0.433 [−1.258, 2.124]
	Memory precision for scenes	0.676 [−0.400, 1.750]	−0.222 [−1.359, 0.915]	0.454 [−1.257, 2.165]	1.201 [−0.151, 2.552]	0.605 [−0.689, 1.898]	−0.406 [−1.587, 0.775]	0.795 [−1.138, 2.728]	0.199 [−1.654, 2.052]
	Memory precision for objects	−1.050 [−2.139, 0.036]	**−1.264** **[−2.338, −0.190]**	**−2.314** **[−3.999, −0.629]**	−0.072 [−1.352, 1.208]	**−1.365** **[−2.59, −0.147]**	**−1.509** **[−2.599, −0.420]**	−1.581 [−3.409, 0.246]	**−2.874** **[−4.625, −1.124]**
	Familiarity-dependent memory	**−1.308** **[−2.224, −0.386]**	−0.195 [−1.109, 0.720]	**−1.503** **[−2.963, −0.043]**	−0.932 [−2.07, 0.221]	**−1.581** **[−2.77, −0.388]**	−0.003 [−0.968, 0.961]	−0.936 [−2.549, 0.677]	−1.584 [−3.285, 0.117]

Unstandardized estimates and 95% confidence intervals are reported. *CU* cognitively unimpaired, *MCI* mild cognitive impairment, *EMMs* estimated marginal means. Estimates in bold are significant at *p* < .050.

The comparison between the CU and MCI groups revealed baseline differences on all tasks, with all MCI patients performing worse than the CU group at baseline. When stratifying the MCI group according to Aβ status, this pattern remained consistent: both MCI sub-groups performed worse than the CU group, with no differences seen between the MCI Aβ− and MCI Aβ+ groups.

Regarding differences in change, the CU and MCI groups showed a difference in familiarity-dependent memory (CSR) performance slope, with the MCI group showing a significantly steeper decline compared to the CU group. The MCI group also showed a significantly negative change in memory precision for objects (MDT-O), but the difference in change between the groups was only marginal, *p* = 0.058 (see Fig. 3). When stratifying the MCI group into MCI Aβ− and MCI Aβ+ groups, differences in change between the CU and the MCI Aβ+ groups were observed in memory precision for objects and familiarity-dependent memory. No differences in change were seen between the CU and MCI Aβ− groups (see Fig. 4). *Post hoc* pairwise comparisons did not reveal any significant differences in change between the MCI Aβ− and MCI Aβ+ groups. See Supplementary Tables 4–11 for full results.

Supplementary Table 12 and the accompanying text detail model comparisons investigating whether the effect of repeated testing differed across cognitive status or study cohort, due to the variations in study design. Indeed, neither cognitive status nor differential exposure to the remote tasks had an effect on the shape of procedural practice effects.

### Short-term change on remote assessments reflects long-term change on in-person neuropsychological tests

We calculated Pearson correlations between model-extracted random slopes on multi-year in-person neuropsychological assessments and slopes on those tasks for which we found group differences in change (MDT-O and CSR). Longitudinal correlations were not significant for memory precision for objects, *r* = −0.05, *p* = 0.573, and were significantly positive for familiarity-dependent memory, *r* = 0.25, *p* = 0.002. The CU group showed a significant change–change correlation between the lPACC and familiarity-dependent memory, *r* = 0.27, *p* = 0.003, while the MCI group did not, *r* = –.04, *p* = .818. Group-wise correlations between change in lPACC and change in memory precision for objects were not significant, CU: *r* = −0.16, *p* = 0.081, MCI: *r* = 0.04, *p* = 0.826 (see Supplementary Fig. 1).

### In-person neuropsychological tests are not sensitive to differences in short-term change

To test whether the lPACC could capture group differences in short-term cognitive change, two lPACC scores within a year of each other were included in an LMM, *N* = 46 (*n*_MCI_ = 25). On average, the measurements were 39.4 weeks apart (range = 8.0 to 52.1 weeks). When comparing the CU and MCI groups, we observed a difference at baseline, *b* = −2.130, 95% CI [−3.018, −1.236], with the MCI group performing worse than the CU group. The groups did not show a difference in change, *b* = −0.025, 95% CI [−0.638, 0.604]. When stratifying the MCI group by Aβ status (*N* = 45; *n*_MCI Aβ− _= 10, *n*_MCI Aβ+_ = 14), group effects were observed at baseline, with the CU group performing better than both the MCI Aβ− group, *b* = −1.766, 95% CI [−2.671, −0.861], and the MCI Aβ+ group, *b* = −2.966, 95% [−3.942, −1.981]. Change in the MCI Aβ− group was not significantly different from change in the CU group, *b* = 0.357, 95% CI [−0.363, 1.077], nor was change in the MCI Aβ+ group, *b* = −0.363, 95% CI [−1.102, 0.415]. See Supplementary Tables 13 and 14 for full results.

Notably, when running the LMMs for the remote tasks in this restricted sample, we observed results consistent with those found in the whole sample. Namely, a significant difference in change between the CU and MCI groups for memory precision for objects was seen, *b* = −1.728, 95% CI [−3.252, −0.154], as well as a marginal difference in change for familiarity-dependent memory, *b* = −1.419, 95% CI [−2.900, 0.079] (*p* = 0.063). Stratifying the MCI group by Aβ status also yielded consistent findings, with the MCI Aβ+ group exhibiting a significant difference in change in memory precision for objects when compared to the CU group, *b* = −2.725, 95% CI [−4.495, −0.977], as well as a marginal difference in change for familiarity-dependent memory, *b* = − 1.605, 95% CI [−3.541, 0.305] (*p* = 0.101).

## Discussion

In the current analyses, we investigated cognitive trajectories in older adults over 30 weeks using frequent remote unsupervised cognitive assessments. First, we established the feasibility of deploying remote and unsupervised cognitive assessments for up to one year. Then, differences between a CU and an MCI group in change on three tests of episodic memory processes were tested, and the effect of Aβ status in the MCI group was explored. Finally, we examined the change–change correlations between remotely measured cognitive performance and cognition measured with established in-person neuropsychological assessments (lPACC) and also tested the sensitivity of in-clinic measurements to differences in short-term cognitive change.

Regarding feasibility, we found that, on average, participants completed 77% of all task sessions, indicating that participants completed most of the assigned remote tasks, and average compliance was 89%, showing that the majority of participants, even those who eventually dropped out of the study, completed the tasks in a timely manner. This is comparable to a previous year-long remote study, which had an adherence rate of 86% in a study design with monthly remote tasks^46^. We found a main effect of study cohort on adherence and compliance; namely, those CU participants from the Wisconsin cohort showed better adherence and compliance than DELCODE participants. This may be because participants from the Wisconsin cohort were contacted if they failed to engage with the study app for a prolonged period of time, while contacting DELCODE participants was not possible. In general, the high adherence and compliance rates indicate that high-frequency remote and unsupervised assessment over up to a year is feasible in older adults with varying degrees of cognitive impairment, though future studies may consider adopting strategies to improve feasibility even further. Encouragingly, we found that neither cognitive status nor amyloid positivity was predictive of drop-out before 30 weeks.

Regarding long-term cognitive change, we found that, compared to the CU group, the MCI group in the current subsample showed decline on established in-person neuropsychological tests over up to eight years, as expected. In terms of short-term change measured remotely, we found that associative memory and memory precision did not show any differences in change between the CU and all-cause MCI groups. However, performance on the CSR (i.e., familiarity-dependent memory) declined in MCI patients compared to CU participants, supporting our hypotheses. Additionally, memory precision for objects and familiarity-dependent memory both showed differences in change between the CU group and the MCI Aβ+ group. This suggests that these tasks, when frequently administered, may be suitable tools to monitor cognitive change in MCI, particularly in amyloid-positive MCI patients, over short periods of time (30 weeks).

Within a moderately sized subsample, we found no evidence that in-person tasks are sensitive to group differences in short-term decline, even when comparing CU individuals to those with an MCI diagnosis and amyloid positivity. Namely, when limiting the analyses to only two measurements per person taken within a year of each other, the lPACC score was not sensitive to differences in change between the CU group and the MCI group, even when stratifying according to Aβ status. This further underlines the need for tools that can capture short-term cognitive changes, either by frequently assessing cognition to improve measurement reliability, administering tasks that are specific to AD-related changes in cognition, or a combination of both approaches. Notably, when restricting the analysis of the remote task performance to the same subsample, we were still able to detect differences in change in memory precision for objects and familiarity-dependent memory between the CU and MCI groups. Altogether, these findings suggest that frequently administered remote and unsupervised cognitive tasks are an appropriate tool to measure subtle cognitive changes that are otherwise undetectable with in-clinic neuropsychological measures.

We found that changes in performance on a familiarity-dependent memory task (CSR) across 30 weeks were correlated with changes in the lPACC measured across eight years. This suggests that the CSR, when frequently administered over a relatively short time frame, captures change in a similar cognitive construct as established in-person neuropsychological tests designed to capture changes due to AD pathology. The MDT-O did not show a significant change–change relationship with the lPACC score when looking at both groups, indicating that the longitudinal trajectories of the underlying constructs measured with these tests might be divergent. This could be due to the lPACC not including any tasks of mnemonic discrimination. Notably, this suggests that novel tests of cognitive processes not traditionally evaluated could provide valuable additional information regarding disease-related changes in cognition.

**Fig. 5:**
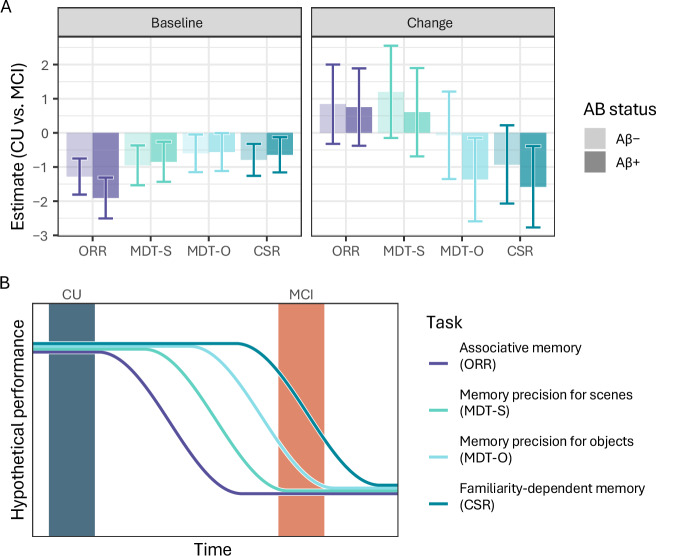
Decline in specific episodic memory processes may be sequential. **A** Estimates of group differences between cognitively unimpaired (CU) and mild cognitive impairment (MCI) groups stratified by Aβ status. Error bars represent 95% confidence intervals. **B** A hypothetical timeline of differential decline in task performance and the time windows of measurement in the current study, in which associative memory measured by the Object-in-Room Recall (ORR) task declines first, followed by the memory precision measured by the Mnemonic Discrimination Task for Scenes (MDT-S) and Objects (MDT-O), respectively, and familiarity-dependent memory measured by the Complex Scene Recognition (CSR) task. The sensitive period for the ORR and MDT-S may be during an earlier disease stage, before MCI can be diagnosed.

Consistent with the hypothesis that decline across the different remote tasks would be sequential rather than simultaneous, we found that memory precision (MDT-OS) and associative memory (ORR) showed baseline group effects that were descriptively larger than those seen for familiarity-dependent memory (CSR). Additionally, we did not observe differences in the 30-week change between groups for the former tasks. We speculate that, while at an earlier point in time, there was a decline in memory precision for scenes and associative memory such that a large group difference was observed at baseline, the rate of decline at the time of measurement was so slow as to be undetectable. That is, the peak rate of decline for memory precision for scenes and associative memory may occur during an earlier disease stage, before a clinical diagnosis of MCI is made. The observed difference in CSR change suggests that MCI might represent a sensitive period for familiarity-dependent memory, such that it is able to be captured within a 30-week window (see Fig. 5).

This explanation is largely consistent with our hypotheses based on the spatiotemporal spread of tau pathology. We expected the MDT-OS and the ORR to show the earliest signs of decline along disease progression, due to their reliance on the hippocampal and entorhinal cortices, in which some of the earliest signs of pathological tau accumulation can be detected^20,21,47,48^. In the current sample, this manifested as more severe impairment in MCI at baseline and a slowed rate of decline. The CSR, in contrast, which relies on extra hippocampal familiarity-dependent memory^38^ and recruits a wide network of brain regions including the medial temporal lobe but also frontal and parietal regions^34–36^, which are typically affected by tau pathology later in the course of disease progression, seems to have been relatively preserved until the MCI stage (i.e., prodromal AD), during which we observed decline. This is also consistent with previous work on familiarity showing impairment only later in disease progression (see ref. ^43^ for a review). Information about tau burden was not available in the current sample, therefore our conclusions in this regard are speculative for now. Future research may directly investigate the effects of regional tau deposition on change in performance in these tasks, as well as declines in performance in preclinical AD samples, in which tau predominantly accumulates in the entorhinal cortex and hippocampus^42^.

Of note, the MCI group did not show floor effects with respect to possible task performance: mean corrected hit rate was 0.334 and 0.387 for the MDT-S and MDT-O, respectively (chance performance = 0), while mean ORR score was 17.7 (71%; chance performance = 33%). Additionally, only 3, 2, and 2% of the MCI group’s scores were below chance for the MDT-S, MDT-O, and CSR, respectively, while no individual performed worse than chance on the ORR at any time point. This further suggests a disease-contingent asymptote (i.e., slowing of decline rate), rather than a psychometric floor effect caused by technical limitations of the tests.

There are a number of limitations that should be considered when interpreting the results of the current analyses. Firstly, this was a harmonized multi-cohort analysis in which the study designs across the three cohorts were not identical; in particular, in-person assessments differed across sites. While this was accounted for by deriving a latent score similar to the PACC, the underlying cognitive construct may have differed slightly across cohorts. Similarly, task schedules varied slightly across cohorts. Parallel task versions were used to minimize practice effects, and both study cohort and session number were included in the models to control for any differences, but future studies may use uniform task schedules to further minimize unwanted effects due to schedule.

Additionally, although the MDT-O and CSR were sensitive to the change in the current MCI sample versus the CU group, 30 weeks may not be a long enough period to see meaningful change in all aspects of episodic memory in early AD, when disease progression is still relatively slow; we may have seen stronger effects across longer time periods. Future studies may consider deploying remote and unsupervised cognitive tests over longer periods of time, and indeed, some studies with such designs are already planned or underway (e.g., REAL AD^49^ and DELCODE 2). Alternatively, the MDT-O and -S in particular had fewer trials than the CSR and ORR, which may have led to lower sensitivity of these tasks over this short time frame, though the fact that we found differences in change in the MDT-O speaks against this.

The MCI group included in the current analyses was also moderately sized, especially considering the stratification according to Aβ status. The generalization of the current results may therefore be limited until they can be replicated in other similar but larger samples.

Another limitation to the current study is that there was no regional tau information available, as neither the DELCODE nor the memory clinic cohorts underwent positron emission tomography (PET) scanning. We also hypothesize that associative memory and memory precision for scenes might detect a decline in performance among individuals who are less advanced in the course of disease progression, such as those who are Aβ positive and experiencing subjective cognitive decline but no objective cognitive impairment, that is, those with preclinical AD (i.e., clinical stage 2 according to recent NIA-AA criteria^16^). However, diagnostic information regarding subjective cognitive decline (SCD) was not available for a large portion of the sample at the time of these analyses.

The results of the current study indicate that disease-relevant cognitive decline can indeed be monitored during a relatively short time frame using frequently administered remote and unsupervised testing. This has implications for the use of remote testing in clinical contexts, namely the use of individualized testing to collect additional information that may inform the diagnostic process, as well as to monitor disease progression and response to treatments. More research with large sample sizes will be required to develop norms in terms of cognitive decline on remote tasks. Once these norms are established, however, patients may be able to complete frequent remote testing using their own devices to measure their individual rate of cognitive decline. A clinician could then compare this to the expected rate of decline for that individual’s demographic group and determine whether they show conspicuous decline, facilitating the diagnostic process and enabling clinicians to better accompany their patients facing progressive memory loss over time. Additionally, scalable cognitive tests that can monitor disease progression and responses to therapies are a necessity in the face of emerging AD treatments. Accessible and high-frequency measurement of cognitive function during treatment could provide additional information to clinicians, enabling more timely and personalized treatment decisions. The non-verbal nature of the cognitive tasks used in the current study is an additional strength, facilitating the large-scale adoption of such remote tests cross-culturally.

In conclusion, we found that (bi-)weekly remote and unsupervised cognitive assessments are feasible in older adults with and without cognitive impairment, showing that these cognitive tests are suitable for high-frequency assessment. This, in turn, improves their measurement reliability compared to yearly tests and thereby increases their sensitivity to subtle cognitive decline. Indeed, remote tests of familiarity-dependent memory and memory precision for objects were sensitive to group differences in cognitive decline, seemingly driven by AD pathology. Additionally, the patterns of change seen on the remote task of familiarity-dependent memory over only 30 weeks reflected a similar pattern of change captured by traditional neuropsychological assessments over multiple years. In-person neuropsychological test scores did not show group differences in short-term change, emphasizing the need for more sensitive assessment tools, for example, those that can be frequently administered. Finally, the remote tasks also showed differential patterns of change, suggesting that they may be sensitive to cognitive changes at different disease stages, though future studies are necessary to confirm this hypothesis. Taken together, the current findings support the use of smartphone- and tablet-based tasks to capture and monitor episodic memory decline in all-cause MCI and prodromal AD.

## Methods

### Participants and human ethics

Volunteers (50 years or older) were recruited for the mobile add-on study through DELCODE^44^, WRAP^45^, and the Wisconsin ADRC, as well as from the local memory clinic. Participants had to own an app-compatible smartphone or tablet with network access and be able to operate the device without assistance. Those with dementia, sensory or motor impairments, or a neurological illness that would impair their ability to complete the tasks were excluded.

All procedures were reviewed and approved by the ethics committees at all involved sites. The University Hospital Magdeburg provided approval for data collection in the DELCODE cohort (217/19; supported by the medical faculties at the University of Bonn, Ludwig Maximilians University Munich, University of Tübingen, Rostock University, University of Göttingen, and University of Cologne), as well as the memory clinic cohort (137/20), and University of Wisconsin-Madison Institutional Review Board provided approval for data collection in the Wisconsin cohort (2018-1246). Protocols were conducted in accordance with the Declaration of Helsinki. Written informed consent was obtained from all individual participants included in the study.

### Cognitive and Aβ status

DELCODE and memory clinic participants were classified as CU without concerns or were diagnosed with SCD or MCI. An SCD diagnosis was given if a patient presented to a memory clinic with memory complaints but scored above −1.5 SD on the subtests of the Consortium to Establish a Registry for Alzheimer’s Disease (CERAD) neuropsychological test battery based on a demographic-normed performance range^44,50^. MCI diagnoses were made based on the current research criteria by the National Institute on Aging and Alzheimer’s Association (NIA-AA)^51,52^. In the current analyses, those with SCD are considered CU, as they show no clinically confirmed objective cognitive impairment. At the beginning of the mobile add-on study, these groups did not differ on the lPACC according to a pairwise comparison of the estimated marginal means, *t* = –0.15, *p* = 0.988. Wisconsin participants were classified as CU or received an MCI diagnosis based on expert consensus, also using NIA-AA criteria^51,52^.

Aβ status was determined with available data closest to the start of the mobile add-on study (before or after) for all participants. For participants recruited through DELCODE, Aβ status was determined using lumbar cerebrospinal fluid (CSF; Mesoscale Diagnostics LLC, Rockville, USA) Aβ_42_/Aβ_40_ ratio (Aβ+ ≤ 0.08). If CSF was unavailable, the individual probability of Aβ positivity in CSF was calculated using age, APOE genotype, and plasma Aβ_42_/Aβ_40_ ratio (Lumipulse panel; Fujirebio Inc., Tokyo, Japan; see ref. ^53^) following ref. ^54^ (Aβ+ > 0.639; determined using the Youden index with a cost ratio of 1.5:1). On average, Aβ measurements were taken 2.7 years before the start of the mobile add-on study (SD = 1.7, range = −6.2 years to 0.0 years).

For participants recruited through the memory clinic, Aβ status was determined using CSF Aβ_42_/Aβ_40_ ratio (Lumipulse panel; Fujirebio Inc., Tokyo, Japan; Aβ+ ≤ 0.69). On average, lumbar punctures were performed 0.3 years before the mobile add-on study (SD = 0.8, range = −3.4 years to 1.1 years).

For participants recruited through WRAP and the Wisconsin ADRC, Aβ status was determined using CSF Aβ_42_/Aβ_40_ ratio (NeuroToolKit panel; Roche Diagnostics International Ltd, Rotkreuz, Switzerland; Aβ+ ≤ 0.046) or [^11^C]Pittsburgh compound B (PiB) PET (Aβ+ ≥ 1.19 DVR) if CSF was not available (see ref. ^55^). On average, Aβ measurements were taken 2.1 years before the mobile add-on study (SD = 3.0, range = −12.0 years to 1.4 years).

### In-clinic neuropsychological assessments

Cognitive test batteries, including tests of global function, category fluency, delayed word and story recall, and executive function, were administered annually or biennially by a clinician or other qualified study team member. On average, the closest in-clinic visit was 0.1 years (SD = 0.1) from the start of the mobile add-on study for DELCODE, 0.4 years (SD = 1.1) for Wisconsin, and less than 0.1 years (SD = 0.3) for the local memory clinic (see Supplementary Fig. 2). Specific tests are described in Table 3 (also see Supplementary Table 15 and Supplementary Fig. 3).

**Table 3:** In-person neuropsychological assessments

Neuropsychological assessment	Description	Administered in which cohorts
Mini-Mental State Examination^63^	30-item screening tool used to assess cognitive status	DELCODE, WRAP, memory clinic
Montreal Cognitive Assessment^64^	10-min cognitive screening tool designed to help general practitioners detect MCI	Wisconsin ADRC (cross-walked to MMSE)
Category fluency: Groceries	Tests semantic memory and executive function	DELCODE, memory clinic
Phonemic fluency	Tests semantic memory and executive function	Wisconsin (C-, F-, and L-words, score divided by 3), memory clinic (S-words)
Category fluency: Animals	Tests semantic memory and executive function	All cohorts
Alzheimer’s Disease Assessment Scale—Cognitive Subscale word recall^65^	10-item word list to assess verbal recall	DELCODE, memory clinic
Free and Cued Selected Reminding Test^66^	A test of free and cued recall of newly learned associations; calculated as freely recalled items × 2 + cued items	DELCODE, memory clinic
Rey Auditory Verbal Learning Test^67^	15-item word list to assess verbal recall	Wisconsin
Symbol Digit Modalities Test^68^	90-item test measuring associative memory, executive function, and processing speed	DELCODE, memory clinic
Trail Making Test B^69^	Tests sustained attention and task switching	All cohorts
Digit Symbol Test^70^	93-item test measuring associative memory, executive function, and processing speed	Wisconsin
Logical Memory Delayed Recall	Tests story recall after a 30-min delay	DELCODE, memory clinic (version B from ref. ^71^); WRAP (version A from ref. ^72^)
Craft Story 21^73^	Tests story recall	Wisconsin ADRC (cross-walked to LMDR A)

*MMSE* mini-mental state examination, *LMDR A* logical memory delayed recall version A.

To harmonize neuropsychological test data across cohorts, we used structural equation modeling with the *lavaan* package^56^. In a first step, a CFA model was built using the baseline scores (i.e., from the chronologically closest in-person visit) as manifest variables, scaled such that the CU group had a mean of 0 and a standard deviation of 1. The loadings of the manifest variables onto a latent variable, lPACC, residual variances (lower bound = 0.001), and residual covariances between fluency tests (due to their similarity) were freely estimated. The variance of lPACC was fixed to 1. The model was run using maximum likelihood (ML) estimation with robust (Huber-White) standard errors (MLR). Missing data were handled using ML estimation. This model showed acceptable fit, CFI = 0.912, RMSEA = 0.069. The standardized loadings from this step were saved (see Supplementary Table 15). In a second step, all data from 4.5 years before to 4.5 years after the start of the mobile add-on study were included in a CFA. The loadings of the manifest variables onto lPACC were fixed to the loadings saved from the first step. The variance of lPACC and residual variances (lower bound = 0.001) were freely estimated and lPACC mean was fixed to 1. The model was run using MLR, and missing data were handled with ML. Each subject’s lPACC score at each time point was extracted and used in the analyses.

### Mobile add-on study

Participants completed remote testing using the *neotivTrials* app (neotiv GmbH) on their own smartphone or tablet for at least 30 weeks. All participants were assisted in downloading the app either in person or via email and phone. Each participant received a pseudonymized ID to use in the app, so that no personal or clinical information was associated with their remote data. All data were transferred to the appropriate research centers in accordance with the General Data Protection Regulation. Remote data were then merged with clinical data before being made available to the principal investigators involved in the current analyses.

Study designs differed slightly across cohorts, but as each study was designed to measure change in the same underlying constructs using the same tasks with identical stimuli, we decided to pool the cohorts to increase statistical power. DELCODE participants and 38 Wisconsin participants were instructed to complete one task every two weeks, while those recruited through the memory clinic completed one task every week. The remaining 53 Wisconsin participants completed all three tasks in a multi-day burst every two months. Parallel task versions were used for each task, such that stimuli were never repeated^57^.

In the MDT-OS, participants saw objects and scenes followed by either an identical repeat image or a similar but not identical lure image. Participants were instructed to either indicate if the current image was identical to the one seen before, or to tap on the part of the image that had changed. In total, 64 pairs were presented, with half of the pairs containing repeated stimuli and half containing lure stimuli. During the first phase (one-back), one pair of images was presented in each trial. After a 24-h (DELCODE, memory clinic, and Wisconsin burst design) or two-week delay (Wisconsin continuous design), the two-back phase, in which two interleaved pairs of images were presented in each trial, could be completed. The second phase was not dependent on the first, thus we assume that the length of the delay between the two phases has a negligible effect on performance and do not correct for this. The median time it took to complete both phases of the MDT was 19.5 min (CU = 18.7, MCI Aβ− = 20.9, MCI Aβ+ = 22.0, *p* = 0.746 as tested with a group-wise ANOVA).

In the ORR, participants saw a series of 25 rooms with two objects presented in each. Participants then saw the empty room with a location indicated by a colored circle and were instructed to indicate which of three objects (target, lure in the correct room but wrong location, or unfamiliar lure) they had seen in that specific location (immediate recall). After a 30-min (DELCODE at odd-numbered sessions and memory clinic), 90-min (Wisconsin), or a 24-h delay (DELCODE at even-numbered sessions), the delayed recall phase, which was identical to the immediate recall phase, could be completed. The median time it took to complete both the immediate and delayed recall phases was 13.4 min (CU = 13.0, MCI Aβ− = 13.8, MCI Aβ+ = 14.7, *p* = 0.622).

In the CSR, participants saw 60 photographic images depicting scenes. During the first phase (encoding), participants were instructed to indicate whether each scene was indoor or outdoor. After a 65-min delay, the second phase (recognition) could be completed, where the encoded images were presented to participants along with 30 new images. They were instructed to indicate whether they had seen the current image before or not, or if they were unsure. The median time it took to complete both encoding and recognition phases was 12.9 min (CU = 12.0, MCI Aβ− = 13.2, MCI Aβ+ = 14.5, *p* = 0.538).

Participants were reminded to complete the tasks via push notifications; they could postpone task completion if the notification came at an inconvenient time (e.g., in case of distraction, fatigue, illness, etc.). They were requested to perform the task in a quiet environment, to put on glasses if necessary, and to adjust the settings of their device so they could clearly see the task stimuli. All task instructions were administered remotely through the app (in German for DELCODE and memory clinic participants, in English for Wisconsin participants), and participants performed all tasks fully unsupervised. After each task, participants were asked if they had been distracted during the task (yes/no), and how well they concentrated during the task (0 to 4, with 4 being the highest). The time that elapsed between the encoding and retrieval phases of the ORR and CSR (time to retrieval) was also recorded during each session.

### Task data processing

To ensure data quality, a number of filtering steps were applied to the task data before analyses. These steps included removing data points that were unusable due to technical problems, prolonged time to retrieval, and excessive timeouts (i.e., unfinished trials). See Supplementary Table 16 for details on how many data points were excluded during the filtering process. After filtering, data were standardized such that the CU group had a mean of 0 and a standard deviation of 1 at baseline. See Supplementary Fig. 4 for correlations between remote tasks at baseline, Supplementary Fig. 5 for raw scores, and Supplementary Fig. 6 for baseline correlations between demographic factors, in-person neuropsychological assessments, and remote task performance.

### Statistical analyses

All data wrangling, statistical analysis, and visualization was done using R in RStudio^58,59^ with the *tidyverse* package^60^. An alpha of 0.05 was used to test for statistical significance. In the current analyses, we were interested in cognitive changes in MCI, particularly in MCI due to AD. Therefore, we included only those CU individuals with healthy Aβ levels (CU Aβ−) as a healthy control group, comparing them to either all MCI patients, or stratifying MCI patients by Aβ burden (MCI Aβ− and MCI Aβ+). Those MCI patients for whom the Aβ status was unknown were excluded from the second set of analyses (*n* = 5); the samples were otherwise identical.

To test the feasibility of high-frequency remote assessments in the current sample, adherence and compliance to the mobile add-on study were calculated for each individual and compared across cognitive status groups and study cohorts. We defined adherence as the number of completed task sessions divided by the maximum number of possible sessions in a given study protocol (percentage of tasks completed). Compliance was calculated as the number of task sessions completed divided by how many tasks an individual could have completed given how long they were active in the mobile add-on study. Linear regression models were used to investigate differences in adherence and compliance across cognitive status (CU and MCI) and study cohort (DELCODE, memory clinic, Wisconsin), taking age, sex, and years of education into account. *Post hoc* pairwise comparisons were done using model-based estimated marginal means with the *emmeans* package^61^.

To establish the presence of cognitive decline within the current MCI group compared to the CU group using gold standard measures, in this case, the harmonized lPACC, LMMs were used to analyze the lPACC scores, controlling for age at the start of the mobile add-on study, sex, years of education, and study cohort (DELCODE, memory clinic, Wisconsin). LMMs, including group (CU or MCI) and all covariates as fixed effects, time spent in the study (centered at each participant’s first remote assessment) as both a fixed and random effect, individual intercept as a random effect, and a group-by-time interaction term were built. Participants with only one time point were excluded from the analyses. The model was estimated using the ML estimator and the BOBYQA optimizer^62^. A second LMM was also run stratifying the MCI group into MCI Aβ− and MCI Aβ+ groups, such that each sub-group was compared to the CU group. Estimated marginal means of within-group intercepts and slopes were compared pairwise using the *emmeans* package^61^.

To address our second aim related to the remote tasks, LMMs were also used to investigate the difference in change in task performance over 30 weeks between CU and MCI groups, controlling for age at the start of the mobile add-on study, sex, years of education, and study cohort (DELCODE, memory clinic, Wisconsin), as well as time to retrieval for ORR and CSR, distraction (yes/no), and concentration (0–4). Additionally, to account for potentially differential effects of exposure to the task across study cohorts, we also controlled for task session (1–10), modeled as a natural spline with *df* = 3. In this way, we model the main effect of chronological time in years, controlling for the effect of repeated testing. Separate models were used for each task (MDT-O, MDT-S, ORR, CSR). Another set of analyses were run, stratifying the MCI group by Aβ status as described above. The same covariates were included, and the same analysis steps were carried out.

To understand the relationship between the change in performance on the remotely assessed cognitive tests and those conducted in person, slope–slope correlations were calculated. Random slopes (i.e., change estimated for each individual) were extracted from LMMs including time as a fixed and random effect and the aforementioned covariates (no group term). Slopes on in-person neuropsychological assessments were correlated with slopes on remote task performance using the Pearson method across groups as well as within each diagnostic group.

Finally, to investigate whether in-person neuropsychological assessments are also sensitive to changes over a shorter time frame (i.e., one year), the lPACC score from the in-person appointments closest to the start of the mobile add-on study as well as the subsequent one (if the two assessments fell within one year of each other) were included in an LMM accounting for age, sex, education, and study cohort, modeling random intercepts.

## Supplementary information

Supplementary Material

## Data Availability

Data used in the preparation of this manuscript may be made available upon reasonable request.

## Code availability

Code used in the preparation of this manuscript may be made available upon reasonable request.
