# Supplementary material for: Smartphone-based detection of subtle memory decline in prodromal Alzheimer’s disease
Source: NPJ Digit Med. 2026 Jun 10;9:402. doi: 10.1038/s41746-026-02731-1 (PMC13254114; doi:10.1038/s41746-026-02731-1)
Supplement: Supplementary file 1 — Supplementary Material [file 41746_2026_2731_MOESM1_ESM.pdf]

Supplementary Material for

Smartphone-based detection of subtle memory decline in prodromal Alzheimer's disease

Polk et al. 2026

Supplementary Results 4

Examples of adherence and compliance to the mobile add-on study 4

Differences in performance in completers vs. dropouts....................4

Supplementary Table 1 | Participant demographics comparing completers vs. dropouts. 5

Model estimates for lPACC linear mixed models 6

Supplementary Table 2 | LMM results for the lPACC comparing CU and MCI groups. 6

Supplementary Table 3 | LMM results for the lPACC comparing CU, MCI Aβ−, and MCI Aβ+ groups. 7

Model estimates for remote task linear mixed models 8

Supplementary Table 4 | Linear mixed model results for the MDT-O comparing CU and MCI groups. 9

Supplementary Table 5 | Linear mixed model results for the MDT-O comparing CU, MCI Aβ−, and MCI Aβ+ groups. 10

Supplementary Table 6 | Linear mixed model results for the MDT-S comparing CU and MCI groups. 11

Supplementary Table 7 | Linear mixed model results for the MDT-S comparing CU, MCI Aβ−, and MCI Aβ+ groups. 12

Supplementary Table 8 | Linear mixed model results for the ORR comparing CU and MCI groups. 13

Supplementary Table 9 | Linear mixed model results for the ORR comparing CU, MCI Aβ−, and MCI Aβ+ groups. 14

Supplementary Table 10 | Linear mixed model results for the CSR comparing CU and MCI groups. 15

Supplementary Table 11 | Linear mixed model results for the CSR comparing CU, MCI Aβ−, and MCI Aβ+ groups. 16

Supplementary Table 12 | Model comparisons exploring differential procedural practice effects between CU and MCI groups and between the various study designs. 17

Slope-slope correlation between in-person and remote tasks....................18

Supplementary Fig. 1 | Correlations between remote cognitive task performance and in-person neuropsychological assessment scores....................18

Model estimates for one-year lPACC linear mixed models 19

Supplementary Table 13 | LMM results for the lPACC comparing CU and MCI groups over one year. 19

Supplementary Table 14 | LMM results for the lPACC comparing CU, MCI Aβ−, and MCI Aβ+ groups. 20

Supplementary Descriptive Information...21

Supplementary Fig. 2 | In-person assessments per subject. Each black diamond indicates an in-person assessment through the parent study or at the memory clinic. 21

Supplementary Table 15 | Information about in-person measurements, including means and standard deviations of in-person neuropsychological tests per cohort at the in-person appointment chronologically closest to the start of the mobile add-on study, as well as loading obtained in the first step of the lPACC estimation. 22

Supplementary Fig. 3 | Raw scores of the in-person neuropsychological assessments as well as the extracted lPACC scores...23

Supplementary Table 16 | Number of observations filtered from each task before analyses. 24

Supplementary Fig. 4 | Pairwise correlations between performance on remote tasks at baseline. 25

Supplementary Fig. 5 | Raw scores on remote tasks over time..........................26

Supplementary Fig. 6 | Pairwise correlations between demographic factors, neuropsychological test scores at the in-person visit chronologically closest to the start of the mobile add-on study, and remote task scores at baseline. 27

Supplementary Results

Examples of adherence and compliance to the mobile add-on study

To calculate adherence, the number of task sessions completed by an individual was divided by the maximum number of task session possible for their cohort, which was as follows:

- DELCODE: 24 task sessions
- Memory clinic: 30 task sessions
- Wisconsin: 18 task sessions

For example, completing 15 task sessions would result in an adherence rate of 63% for DELCODE participants, 50% for memory clinic participants, and 83% for Wisconsin participants.

To calculate compliance, first, the time spent in the study was calculated for each individual based on the last task they completed. Then, the possible number of task sessions that could have been completed during this time frame was calculated based on the interval of testing, which was as follows:

- DELCODE: One task session every 14 days (i.e., every other week)
- Memory clinic: One task session every 7 days (i.e., once a week)
- Wisconsin: One task session every 20 days (i.e., three task sessions every two months)

For example, an individual in DELCODE whose last task session was completed on day 100 could have completed 8 tasks in this time frame (on days 0, 14, 28, 42, 56, 70, 84, and 98). If they completed 6 tasks in this time, they would have a compliance rate of 6 / 8 = 75%. An individual from the memory clinic whose last task session was completed on day 100 could have completed 15 tasks (on days 0, 7, 14, 21, 28, 35, 42, 49, 56, 63, 70, 77, 84, 91, and 98). If they completed 6 tasks in this time, they would have a compliance rate of 6 / 15 = 40%. Finally, if an individual from the Wisconsin cohort whose last task session was completed on day 100 could have completed 6 tasks (on days 0, 20, 40, 60, 80, and 100). If they completed 6 tasks in this time, they would have a compliance rate of 6 / 6 = 100%.

Differences in performance in completers vs. dropouts

In this set of analyses, we analyzed data collected over up to 30 weeks of the mobile add-on study. A logistic regression model was used to evaluate the effect of demographic factors, including cognitive and Aβ status, on drop-out (coded as 0 = did not drop-out, 1 = dropped out by week 30). Statistics are shown in Supplementary Table 1. Younger age at the start of the mobile add-on study, being a part of the Wisconsin cohort, and female sex were associated with a lower probability of dropping out. Neither cognitive nor Aβ status were associated with drop-out.

Supplementary Table 1 | Participant demographics comparing completers vs. dropouts.

	All	Completed	Dropped out by week 30	Effect on drop-out OR (*p*-value)
Participants	202	147 (73%)	55 (27%)	
Cognitive status				
CU	152	122 (83%)	30 (55%)	
MCI	50	25 (17%)	25 (45%)	0.95 (.935)
Aβ status	5 missing			
Aβ−	173 (86%)	134 (91%)	39 (71%)	
Aβ+	24 (12%)	11 (7%)	13 (24%)	1.52 (.524)
Study cohort				
DELCODE	77 (38%)	51 (35%)	26 (47%)	
Memory clinic	34 (17%)	15 (10%)	19 (35%)	2.18 (.197)
Wisconsin	91 (45%)	81 (55%)	10 (18%)	0.31 (.011)
Age at start of mobile add-on study (years)	69.9 ± 6.8	69.2 ± 6.4	71.8 ± 7.5	1.06 (.034)
Sex (female)	118 (58%)	96 (65%)	22 (40%)	0.45 (.031)
Education (years)	15.4 ± 2.7	15.6 ± 2.8	14.8 ± 2.4	0.98 (.794)

OR = odds ratio, CU = cognitively unimpaired, MCI = mild cognitive impairment. *N*s and percentages of all participants in the (sub-) sample are reported for Participants, Cognitive status, Aβ status, Study cohort, and Sex. Means and standard deviations are reported for Age and Education.
CU, Aβ−, DELCODE, and male sex served as reference groups in the logistic regression. Intercept: OR = 0.01, *p* = .061.

Additionally, we tested whether drop-out was associated with baseline performance on the remote tasks. Linear regression models including main effects of drop-out and cognitive status, as well as their interaction effect, and demographic and contextual factors (age, sex, years of education, study cohort, distraction, concentration, time of day, and time to retrieval [for ORR and CSR]) as covariates of no interest. This revealed a significant interaction effect on MDT-O baseline performance: the difference between completers and drop-outs was greater in the MCI group than the CU group, $b = 0.990$, $p = .025$, with MCI participants who dropped out performing worse than those who completed the study up to 30 weeks. Therefore, to ensure that our results regarding group differences in change in MDT-O were not affected by participant retention, a sensitivity analysis was run excluding those participants who dropped out before 30 weeks. We found an identical pattern of results; namely, when comparing the CU and MCI groups, we found a marginally more negative trajectory for the MCI group, $b = -1.241$, $p = .057$, and when stratifying the MCI group by Aβ status, we found no difference in change between the CU and MCI Aβ− groups, $b = -0.467$, $p = .522$, and a significantly greater decline in the MCI Aβ+ group versus the CU group, $b = -1.757$, $p = .021$.

Model estimates for lPACC linear mixed models

Differences in change between CU and MCI groups were investigated using linear mixed models (LMMs):

$$\text{score} \sim \text{group} \times \text{years} + (\text{years} \mid \text{id}) + \text{age} + \text{sex} + \text{years of education} + \text{study cohort}$$

Regarding the main effects of interest, scores were centered to the CU group such that they had a mean of 0 and standard deviation (SD) of 1 at baseline. The unstandardized estimate (*b*) for the group term can be interpreted as the difference between groups in SD units relative to the CU group. As time in years was included, *b* for the years term can be interpreted as annual rate of change in SD units relative to the CU group, and *b* for the group-by-years interaction term can be interpreted as the differences in the annual rate of change between groups.

Age and years of education were simply centered, therefore *b* can be interpreted as the difference in score in SD units relative to the CU group for every 1 SD change in the predictor. Sex and study cohort were included as factors, therefore *b* can be interpreted as the difference in score in SD units relative to the CU group when compared to the reference group (group [CU], sex [male], study cohort [DELCODE]).

Supplementary Table 2 | LMM results for the lPACC comparing CU and MCI groups.

Parameter	Unstandardized estimate (*b*)	*p*-value
Intercept	−0.442 [−0.716, −0.169]	**.002**
**Group [MCI]**	−1.986 [−2.422, −1.554]	**< .001**
**Years**	−0.026 [−0.053, 0.001]	.057
**Years * Group [MCI]**	−0.198 [−0.258, −0.139]	**< .001**
Age	−0.204 [−0.338, −0.070]	**.003**
Sex [female]	0.727 [0.464, 0.990]	**< .001**
Years of education	0.301 [0.161, 0.440]	**< .001**
Study cohort [Memory clinic]	−1.169 [−1.661, −0.673]	**< .001**
Study cohort [Wisconsin]	−0.136 [−0.436, 0.163]	.366

		Group-wise estimated marginal means
Group	CU	−0.564 [−0.791, −0.338]
	MCI	−2.551 [−2.862, −2.239]
Years * Group	CU	−0.026 [−0.053, 0.001]
	MCI	−0.224 [−0.277, −0.171]

N = 191, 861 observations included in the model. Parameters in bold are discussed in the main text. *P*-values in bold are significant at $p < .050$ uncorrected. CU = cognitively unimpaired, MCI = mild cognitive impairment.

Supplementary Table 3 | LMM results for the lPACC comparing CU, MCI Aβ−, and MCI Aβ+ groups.

Parameter	Unstandardized estimate (*b*)	*p*-value
Intercept	−0.401 [−0.665, −0.139]	**.003**
**Group [MCI Aβ−]**	−1.529 [−2.002, −1.058]	**< .001**
**Group [MCI Aβ+]**	−2.583 [−3.101, −2.068]	**< .001**
**Years**	−0.025 [−0.050, −0.001]	**.042**
**Years * Group [MCI Aβ−]**	−0.066 [−0.140, 0.006]	.075
**Years * Group [MCI Aβ+]**	−0.337 [−0.412, −0.263]	**< .001**
Age	−0.207 [−0.338, −0.076]	**.002**
Sex [female]	0.682 [0.423, 0.940]	**< .001**
Years of education	0.297 [0.158, 0.435]	**< .001**
Study cohort [Memory clinic]	−1.187 [−1.678, −0.691]	**< .001**
Study cohort [Wisconsin]	−0.152 [−0.446, 0.142]	.304

		Group-wise estimated marginal means	Difference from MCI Aβ−
Group	CU	−0.552 [−0.773, −0.332]	
	MCI Aβ−	−2.082 [−2.484, −1.679]	
	MCI Aβ+	−3.135 [−3.549, −2.721]	−1.053 [−1.716, −0.391]
Years * Group	CU	−0.025 [−0.050, 0.000]	
	MCI Aβ−	−0.091 [−0.161, −0.021]	
	MCI Aβ+	−0.362 [−0.433, −0.290]	−0.270 [−0.389, −0.151]

N = 187, 846 observations included in the model. Parameters in bold are discussed in the main text. *P*-values in bold are significant at $p < .050$ uncorrected. CU = cognitively unimpaired, MCI Aβ− = mild cognitive impairment without amyloid pathology, MCI Aβ+ = mild cognitive impairment with amyloid pathology.

Model estimates for remote task linear mixed models

Differences in change between CU and MCI groups were investigated using linear mixed models (LMMs):

$$\text{score} \sim \text{group} \times \text{years} + (\text{years} \mid \text{id}) + \text{age} + \text{sex} + \text{years of education} + \text{study cohort} + \underline{ns}(\text{task session}, df = 3) + \text{concentration} + \text{distraction} + \text{time of day}$$

Regarding the main effects of interest, scores were again centered to the CU group such that they had a mean of 0 and SD of 1 at baseline. The *b* can therefore be interpreted as described above.

Age, years of education, and concentration were simply centered, therefore *b* can be interpreted as the difference in score in SD units relative to the CU group for every 1 SD change in the predictor. Sex, study cohort, distraction, and time of day were included as factors, therefore *b* can be interpreted as the difference in score in SD units relative to the CU group when compared to the reference group (group [CU], sex [male], study cohort [DELCODE], distraction [no], time of day [6AM to 12PM]). The effect of repeated testing was modeled with a natural spline with 3 degrees of freedom. The individual *b*s are uninterpretable on their own, therefore a likelihood ratio test comparing models including and excluding this term was conducted and the *p*-value is reported.

Supplementary Table 4 | Linear mixed model results for the MDT-O comparing CU and MCI groups.

Parameter	Unstandardized estimate (*b*)	*p*-value	η^2_p
Intercept	0.174 [−0.148, 0.497]	.288	
**Group [MCI]**	−0.537 [−1.015, −0.059]	**.028**	.025
**Years**	−1.264 [−2.327, −0.203]	**.019**	.019
**Years * Group [MCI]**	−1.050 [−2.139, 0.036]	.058	.016
Age	−0.228 [−0.352, −0.103]	**< .001**	.072
Sex [female]	0.005 [−0.242, 0.250]	.970	.000
Years of education	−0.017 [−0.143, 0.109]	.792	.000
Study cohort [Memory clinic]	−0.556 [−1.034, −0.077]	**.023**	.026
Study cohort [Wisconsin]	−0.008 [−0.289, 0.273]	.958	
Task session (natural spline)		**.038**	.020
Concentration	−0.002 [−0.080, 0.075]	.952	.000
Distraction [yes]	−0.122 [−0.364, 0.118]	.318	.002
Time of day [12PM to 6PM]	−0.137 [−0.298, 0.024]	.096	
Time of day [6PM to 12AM]	−0.252 [−0.437, −0.068]	**.007**	.013
Time of day [12AM to 6AM]	−0.448 [−0.978, 0.079]	.094	

		Group-wise estimated marginal means
Group	CU	0.153 [−0.232, 0.537]
	MCI	−0.384 [−0.924, 0.156]
Years * Group	CU	−1.264 [−2.338, −0.190]
	MCI	−2.314 [−3.999, −0.629]

N = 170, 721 observations included in the model. Parameters in bold are discussed in the main text. *P*-values in bold are significant at *p* < .050 uncorrected. CU = cognitively unimpaired, MCI = mild cognitive impairment.

Supplementary Table 5 | Linear mixed model results for the MDT-O comparing CU, MCI Aβ−, and MCI Aβ+ groups.

Parameter	Unstandardized estimate (*b*)	*p*-value	η^2_p
Intercept	0.160 [−0.162, 0.482]	.329	
**Group [MCI Aβ−]**	−0.600 [−1.151, −0.047]	**.033**	.037
**Group [MCI Aβ+]**	−0.562 [−1.118, −0.008]	**.047**	
**Years**	−1.509 [−2.580, −0.440]	**.006**	.023
**Years * Group [MCI Aβ−]**	−0.072 [−1.352, 1.208]	.912	.036
**Years * Group [MCI Aβ+]**	−1.365 [−2.590, −0.147]	**.029**	
Age	−0.241 [−0.367, −0.116]	**< .001**	.078
Sex [female]	0.007 [−0.243, 0.255]	.957	.000
Years of education	0.000 [−0.128, 0.128]	.995	.000
Study cohort [Memory clinic]	−0.609 [−1.090, −0.126]	**.014**	.032
Study cohort [Wisconsin]	0.003 [−0.278, 0.284]	.982	
Task session (natural spline)		**.001**	.042
Concentration	0.001 [−0.077, 0.080]	.973	.000
Distraction [yes]	−0.107 [−0.351, 0.136]	.386	.001
Time of day [12PM to 6PM]	−0.141 [−0.304, 0.022]	.090	
Time of day [6PM to 12AM]	−0.257 [−0.445, −0.068]	**.008**	.014
Time of day [12AM to 6AM]	−0.466 [−0.992, 0.058]	.080	

		Group-wise estimated marginal means	Difference from MCI Aβ−
Group	CU	0.195 [−0.188, 0.578]	
	MCI Aβ−	−0.405 [−1.012, 0.202]	
	MCI Aβ+	−0.367 [−0.982, 0.247]	0.038 [−0.698, 0.774]
Years * Group	CU	−1.509 [−2.599, −0.420]	
	MCI Aβ−	−1.581 [−3.409, 0.246]	
	MCI Aβ+	−2.874 [−4.625, −1.124]	−1.293 [−3.125, 0.539]

N = 165, 686 observations included in the model. Parameters in bold are discussed in the main text. *P*-values in bold are significant at $p < .050$ uncorrected. CU = cognitively unimpaired, MCI Aβ− = mild cognitive impairment without amyloid pathology, MCI Aβ+ = mild cognitive impairment with amyloid pathology.

Supplementary Table 6 | Linear mixed model results for the MDT-S comparing CU and MCI groups.

Parameter	Unstandardized estimate (*b*)	*p*-value	η^2_p
Intercept	−0.045 [−0.388, 0.298]	.798	
**Group [MCI]**	−0.844 [−1.337, −0.354]	**.001**	.038
**Years**	−0.222 [−1.348, 0.903]	.698	.000
**Years * Group [MCI]**	0.676 [−0.400, 1.750]	.217	.002
Age	−0.244 [−0.376, −0.111]	**< .001**	070
Sex [female]	−0.284 [−0.547, −0.021]	.035	.025
Years of education	−0.046 [−0.181, 0.089]	.502	.003
Study cohort [Memory clinic]	−0.216 [−0.717, 0.286]	**.396**	.009
Study cohort [Wisconsin]	0.150 [−0.151, 0.450]	.326	
Task session (natural spline)		**< .001**	.069
Concentration	0.046 [−0.039, 0.130]	.291	.002
Distraction [yes]	−0.219 [−0.485, 0.046]	.105	.004
Time of day [12PM to 6PM]	0.095 [−0.084, 0.273]	.297	
Time of day [6PM to 12AM]	0.057 [−0.148, 0.261]	.584	.002
Time of day [12AM to 6AM]	0.200 [−0.383, 0.782]	.501	

		Group-wise estimated marginal means
Group	CU	−0.248 [−0.667, 0.171]
	MCI	−1.092 [−1.656, −0.528]
Years * Group	CU	−0.222 [−1.359, 0.915]
	MCI	0.454 [−1.257, 2.165]

N = 170, 721 observations included in the model. Parameters in bold are discussed in the main text. *P*-values in bold are significant at *p* < .050 uncorrected. CU = cognitively unimpaired, MCI = mild cognitive impairment.

Supplementary Table 7 | Linear mixed model results for the MDT-S comparing CU, MCI Aβ−, and MCI Aβ+ groups.

Parameter	Unstandardized estimate (*b*)	*p*-value	η^2_p
Intercept	−0.003 [−0.350, 0.343]	.986	
**Group [MCI Aβ−]**	−0.950 [−1.533, −0.368]	**.001**	.043
**Group [MCI Aβ+]**	−0.847 [−1.434, −0.262]	**.005**	
**Years**	−0.406 [−1.574, 0.759]	**.493**	.000
**Years * Group [MCI Aβ−]**	1.201 [−0.151, 2.552]	.081	.005
**Years * Group [MCI Aβ+]**	0.605 [−0.689, 1.898]	.358	
Age	−0.229 [−0.364, −0.094]	**.001**	.060
Sex [female]	−0.289 [−0.558, −0.020]	**.036**	.025
Years of education	−0.060 [−0.197, 0.078]	.395	.004
Study cohort [Memory clinic]	−0.252 [−0.767, 0.263]	.336	.011
Study cohort [Wisconsin]	0.161 [−0.143, 0.463]	.297	
Task session (natural spline)		**< .001**	.074
Concentration	0.035 [−0.051, 0.120]	. 424	.001
Distraction [yes]	−0.198 [−0.465, 0.069]	. 146	.003
Time of day [12PM to 6PM]	0.092 [−0.088, 0.270]	.315	.003
Time of day [6PM to 12AM]	−0.013 [−0.220, 0.194]	.902	
Time of day [12AM to 6AM]	0.186 [−0.389, 0.760]	.526	

		Group-wise estimated marginal means	Difference from MCI Aβ−
Group	CU	−0.173 [−0.591, 0.245]	
	MCI Aβ−	−1.123 [−1.768, −0.477]	
	MCI Aβ+	−1.020 [−1.673, −0.367]	0.103 [−0.665, 0.871]
Years * Group	CU	−0.406 [−1.587, 0.775]	
	MCI Aβ−	0.795 [−1.138, 2.728]	
	MCI Aβ+	0.199 [−1.654, 2.052]	−0.596 [−2.476, 1.283]

N = 165, 686 observations included in the model. Parameters in bold are discussed in the main text. *P*-values in bold are significant at $p < .050$ uncorrected. CU = cognitively unimpaired, MCI Aβ− = mild cognitive impairment without amyloid pathology, MCI Aβ+ = mild cognitive impairment with amyloid pathology.

Supplementary Table 8 | Linear mixed model results for the ORR comparing CU and MCI groups.

Parameter	Unstandardized estimate (*b*)	*p*-value	η^2_p
Intercept	−0.372 [−0.693, −0.051]	**.024**	
**Group [MCI]**	−1.443 [−1.911, −0.975]	**< .001**	.184
**Years**	−0.240 [−1.184, 0.704]	.618	.000
**Years * Group [MCI]**	0.587 [−0.382, 1.553]	.229	.009
Age	−0.212 [−0.341, −0.083]	**.001**	.061
Sex [female]	0.127 [−0.132, 0.387]	.332	.006
Years of education	−0.002 [−0.130, 0.128]	.980	.000
Study cohort [Memory clinic]	−0.144 [−0.665, 0.380]	.588	.004
Study cohort [Wisconsin]	0.089 [−0.204, 0.383]	.545	
Task session (natural spline)		**.041**	.022
Time to retrieval	−0.306 [−0.357, −0.255]	**< .001**	.192
Concentration	0.264 [0.180, 0.347]	**< .001**	.056
Distraction [yes]	−0.315 [−0.540, −0.090]	**.006**	.012
Time of day [12PM to 6PM]	0.10 [−0.042, 0.249]	.161	.005
Time of day [6PM to 12AM]	0.056 [−0.095, 0.207]	.467	
Time of day [12AM to 6AM]	−0.140 [−0.610, 0.330]	.558	

		Group-wise estimated marginal means
Group	CU	0.035 [−0.339, 0.409]
	MCI	−1.408 [−1.923, −0.894]
Years * Group	CU	−0.240 [−1.203, 0.722]
	MCI	0.347 [−1.217, 1.910]

N = 162, 706 observations included in the model. Parameters in bold are discussed in the main text. *P*-values in bold are significant at $p < .050$ uncorrected. CU = cognitively unimpaired, MCI = mild cognitive impairment.

Supplementary Table 9 | Linear mixed model results for the ORR comparing CU, MCI Aβ−, and MCI Aβ+ groups.

Parameter	Unstandardized estimate (*b*)	*p*-value	η^2_p
Intercept	−0.377 [−0.696, −0.060]	**.020**	
**Group [MCI Aβ−]**	−1.281 [−1.808, −0.752]	**< .001**	.246
**Group [MCI Aβ+]**	−1.907 [−2.507, −1.310]	**< .001**	
**Years**	−0.318 [−1.291, 0.648]	.515	.000
**Years * Group [MCI Aβ−]**	0.843 [−0.332, 2.000]	. 153	.027
**Years * Group [MCI Aβ+]**	0.752 [−0.377, 1.889]	.191	
Age	−0.231 [−0.360, −0.102]	**.001**	.073
Sex [female]	0.118 [−0.142, 0.377]	.367	.005
Years of education	−0.016 [−0.114, 0.146]	.809	.000
Study cohort [Memory clinic]	−0.164 [−0.699, 0.375]	.547	.005
Study cohort [Wisconsin]	0.084 [−0.205, 0.375]	.560	
Task session (natural spline)		**.027**	.023
Time to retrieval	−0.307 [−0.358, −0.256]	**< .001**	.203
Concentration	0.252 [0.169, 0.336]	**< .001**	.054
Distraction [yes]	−0.321 [−0.544, −0.099]	**.005**	.014
Time of day [12PM to 6PM]	0.124 [−0.022, 0.270]	.096	
Time of day [6PM to 12AM]	0.054 [−0.099, 0.206]	.489	.007
Time of day [12AM to 6AM]	−0.159 [−0.627, 0.307]	.502	

		Group-wise estimated marginal means	Difference from MCI Aβ−
Group	CU	0.041 [−0.331, 0.414]	
	MCI Aβ−	−1.239 [−1.823, −0.656]	
	MCI Aβ+	−1.866 [−2.498, −1.233]	−0.626 [−1.420, 0.167]
Years * Group	CU	−0.318 [−1.301, 0.664]	
	MCI Aβ−	0.525 [−1.164, 2.213]	
	MCI Aβ+	0.433 [−1.238, 2.124]	−0.092 [−1.728, 1.545]

N = 157, 672 observations included in the model. Parameters in bold are discussed in the main text. *P*-values in bold are significant at $p < .050$ uncorrected. CU = cognitively unimpaired, MCI Aβ− = mild cognitive impairment without amyloid pathology, MCI Aβ+ = mild cognitive impairment with amyloid pathology.

Supplementary Table 10 | Linear mixed model results for the CSR comparing CU and MCI groups.

Parameter	Unstandardized estimate (*b*)	*p*-value	η^2_p
Intercept	−0.561 [−0.868, −0.254]	**.001**	
**Group [MCI]**	−0.662 [−1.083, −0.240]	**.002**	.048
**Years**	−0.195 [−1.093, 0.710]	.671	.008
**Years * Group [MCI]**	−1.308 [−2.224, −0.386]	**.006**	.046
Age	−0.050 [−0.172, 0.073]	.424	.004
Sex [female]	0.274 [0.025, 0.522]	**.029**	.026
Years of education	0.115 [−0.009, 0.241]	.070	.018
Study cohort [Memory clinic]	−0.059 [−0.519, 0.399]	.800	.025
Study cohort [Wisconsin]	0.304 [0.027, 0.578]	**.029**	
Task session (natural spline)		**.049**	.021
Time to retrieval	−0.283 [−0.350, −0.217]	**< .001**	.093
Concentration	0.153 [0.067, 0.240]	**< .001**	.017
Distraction [yes]	−0.251 [−0.511, 0.009]	.057	.006
Time of day [12PM to 6PM]	0.178 [0.036, 0.319]	**.014**	.019
Time of day [6PM to 12AM]	0.078 [−0.069, 0.224]	.294	
Time of day [12AM to 6AM]	−0.284 [−0.624, 0.055]	.101	

		Group-wise estimated marginal means
Group	CU	−0.192 [−0.540, 0.156]
	MCI	−0.854 [−1.327, −0.382]
Years * Group	CU	−0.195 [−1.109, 0.720]
	MCI	−1.503 [−2.963, −0.043]

N = 174, 744 observations included in the model. Parameters in bold are discussed in the main text. *P*-values in bold are significant at *p* < .050 uncorrected. CU = cognitively unimpaired, MCI = mild cognitive impairment.

Supplementary Table 11 | Linear mixed model results for the CSR comparing CU, MCI Aβ−, and MCI Aβ+ groups.

Parameter	Unstandardized estimate (*b*)	*p*-value	η^2_p
Intercept	−0.534 [−0.839, −0.230]	**.001**	
**Group [MCI Aβ−]**	−0.790 [−1.259, −0.322]	**.001**	.069
**Group [MCI Aβ+]**	−0.643 [−1.158, −0.127]	**.015**	
**Years**	−0.003 [−0.947, 0.947]	**.994**	.007
**Years * Group [MCI Aβ−]**	−0.932 [−2.070, 0.221]	.110	.054
**Years * Group [MCI Aβ+]**	−1.581 [−2.770, −0.388]	**.010**	
Age	−0.067 [−0.188, 0.055]	.278	.007
Sex [female]	0.289 [0.044, 0.534]	**.020**	.030
Years of education	0.124 [−0.001, 0.248]	.052	.022
Study cohort [Memory clinic]	−0.216 [−0.684, 0.248]	.360	.031
Study cohort [Wisconsin]	0.289 [0.019, 0.558]	**.034**	
Task session (natural spline)		**.032**	.025
Time to retrieval	−0.268 [−0.337, −0.199]	**< .001**	.082
Concentration	0.140 [0.051, 0.229]	**.002**	.014
Distraction [yes]	−0.346 [−0.620, −0.073]	**.012**	.010
Time of day [12PM to 6PM]	0.192 [0.044, 0.338]	**.010**	
Time of day [6PM to 12AM]	0.095 [−0.057, 0.246]	.217	.020
Time of day [12AM to 6AM]	−0.270 [−0.615, 0.074]	.123	

		Group-wise estimated marginal means	Difference from MCI Aβ−
Group	CU	−0.365 [−0.721, −0.010]	
	MCI Aβ−	−1.156 [−1.695, −0.616]	
	MCI Aβ+	−1.009 [−1.572, −0.445]	0.147 [−0.515, 0.809]
Years * Group	CU	−0.003 [−0.968, 0.961]	
	MCI Aβ−	−0.936 [−2.549, 0.677]	
	MCI Aβ+	−1.584 [−3.285, 0.117]	−0.649 [−2.387, 1.089]

N = 169, 708 observations included in the model. Parameters in bold are discussed in the main text. *P*-values in bold are significant at $p < .050$ uncorrected. CU = cognitively unimpaired, MCI Aβ− = mild cognitive impairment without amyloid pathology, MCI Aβ+ = mild cognitive impairment with amyloid pathology.

Supplementary Table 12 | Model comparisons exploring differential procedural practice effects between CU and MCI groups and between the various study designs.

Comparison	Task	χ^2 of difference in models
Model 1 vs. Model 2	ORR	χ^2_3 = 6.25, p = .100
	MDT-O	χ^2_3 = 5.16, p = .161
	MDT-S	χ^2_3 = 5.34, p = .149
	CSR	χ^2_3 = 6.42, p = .093
Model 1 vs. Model 3	ORR	χ^2_6 = 10.34, p = .111
	MDT-O	χ^2_6 = 5.16, p = .523
	MDT-S	χ^2_6 = 8.62, p = .196
	CSR	χ^2_6 = 8.68, p = .192

Model 1: Score ~ cognitive status [CU/MCI] × ns(session, *df* = 3) + study design [DELCODE/memory clinic/Wisconsin] × ns(session, *df* = 3) + (session | id) + years + age + sex + years of education + time to retrieval [for ORR and CSR] + distraction [yes/no] + concentration + time of day [morning, afternoon, evening, night]
Model 2: Score ~ **cognitive status [CU/MCI] + ns(session, *df* = 3)** + study design [DELCODE/memory clinic/Wisconsin] × ns(session, *df* = 3) + (session | id) + years + age + sex + years of education + time to retrieval [for ORR and CSR] + distraction [yes/no] + concentration + time of day [morning, afternoon, evening, night]
Model 3: Score ~ cognitive status [CU/MCI] × ns(session, *df* = 3) + **study design [DELCODE/memory clinic/Wisconsin] + ns(session, *df* = 3)** + (session | id) + years + age + sex + years of education + time to retrieval [for ORR and CSR] + distraction [yes/no] + concentration + time of day [morning, afternoon, evening, night]

Slope-slope correlation between in-person and remote tasks

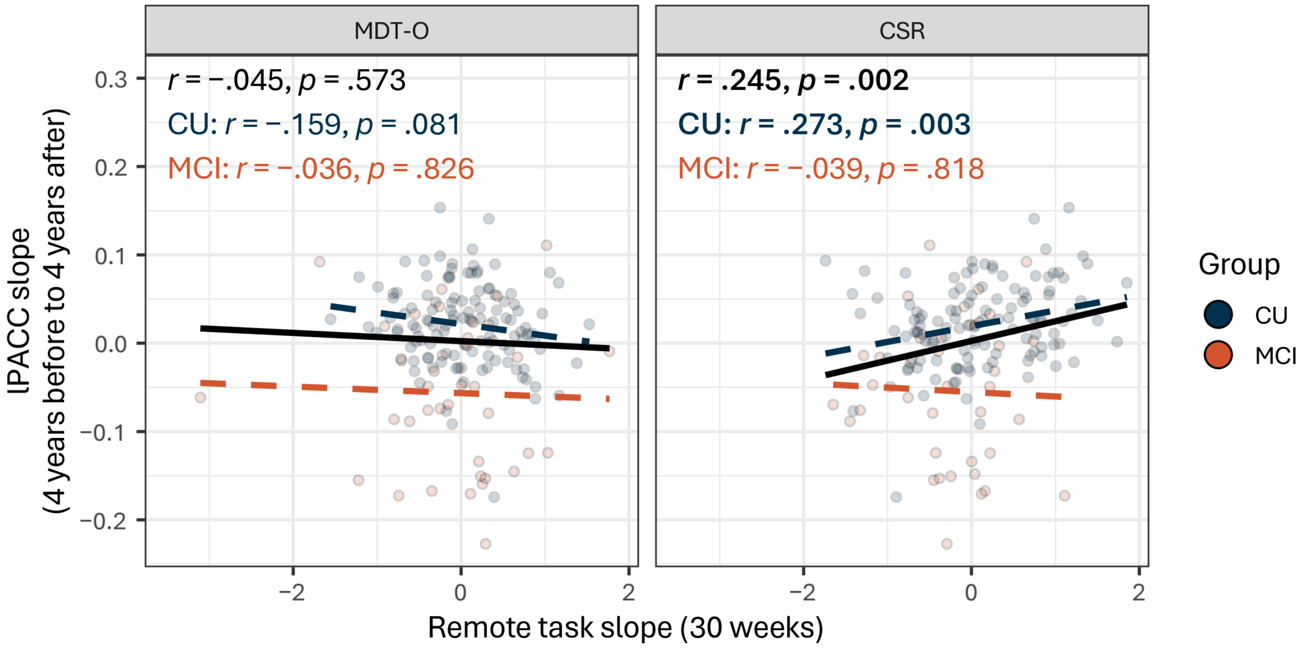

Supplementary Fig. 1 | Correlations between remote cognitive task performance and in-person neuropsychological assessment scores.

Pearson correlations between estimated individual change on remote task performance (MDT-O and CSR only) and annual lPACC slope estimated with data from 4.5 years before and after the start of the mobile add-on study. Change in CSR showed a positive correlation with change in lPACC.
MDT-O = Mnemonic Discrimination Task for Objects; CSR = Complex Scene Recognition.

Model estimates for one-year lPACC linear mixed models

Supplementary Table 13 | LMM results for the lPACC comparing CU and MCI groups over one year.

Parameter	Unstandardized estimate (*b*)	*p*-value
Intercept	0.261 [−0.407, 0.929]	.439
**Group [MCI]**	−2.130 [−3.018, −1.236]	**< .001**
**Years**	0.011 [−0.408, 0.431]	.957
**Years * Group [MCI]**	−0.025 [−0.638, 0.604]	.937
Age	−0.218 [−0.584, 0.149]	.240
Sex [female]	0.759 [−0.053, 1.571]	.068
Years of education	0.556 [0.111, 1.001]	**.016**
Study cohort [Memory clinic]	−1.902 [−2.812, −0.992]	**< .001**

		Group-wise estimated marginal means
Group	CU	−0.602 [−1.283, 0.079]
	MCI	−2.732 [−3.297, −2.166]
Years * Group	CU	0.011 [−0.420, 0.442]
	MCI	−0.013 [−0.482, 0.455]

N = 46, 92 observations included in the model. Parameters in bold are discussed in the main text. *P*-values in bold are significant at $p < .050$ uncorrected. CU = cognitively unimpaired, MCI = mild cognitive impairment.

Supplementary Table 14 | LMM results for the lPACC comparing CU, MCI Aβ−, and MCI Aβ+ groups.

Parameter	Unstandardized estimate (*b*)	*p*-value
Intercept	0.438 [−0.158, 1.034]	.148
**Group [MCI Aβ−]**	−1.766 [−2.671, −0.861]	**< .001**
**Group [MCI Aβ+]**	−2.966 [−3.942, −1.981]	**< .001**
**Years**	0.017 [−0.381, 0.416]	**.931**
**Years * Group [MCI Aβ−]**	0.357 [−0.363, 1.077]	.326
**Years * Group [MCI Aβ+]**	−0.363 [−1.102, 0.415]	.336
Age	−0.267 [−0.590, 0.058]	.106
Sex [female]	0.191 [−0.578, 0.960]	.621
Years of education	0.361 [−0.042, 0.763]	.079
Study cohort [Memory clinic]	−1.637 [−2.485, −0.789]	**< .001**

		Group-wise estimated marginal means	Difference from MCI Aβ−
Group	CU	−0.501 [−1.120, 0.118]	
	MCI Aβ−	−2.266 [−3.040, −1.493]	
	MCI Aβ+	−3.467 [−4.213, −2.721]	−1.200 [−2.544, 0.143]
Years * Group	CU	0.017 [−0.396, 0.431]	
	MCI Aβ−	0.374 [−0.250, 0.998]	
	MCI Aβ+	−0.345 [−1.004, 0.314]	−0.720 [−1.811, 0.372]

N = 45, 90 observations included in the model. Parameters in bold are discussed in the main text. *P*-values in bold are significant at $p < .050$ uncorrected. CU = cognitively unimpaired, MCI Aβ− = mild cognitive impairment without amyloid pathology, MCI Aβ+ = mild cognitive impairment with amyloid pathology.

Supplementary Descriptive Information

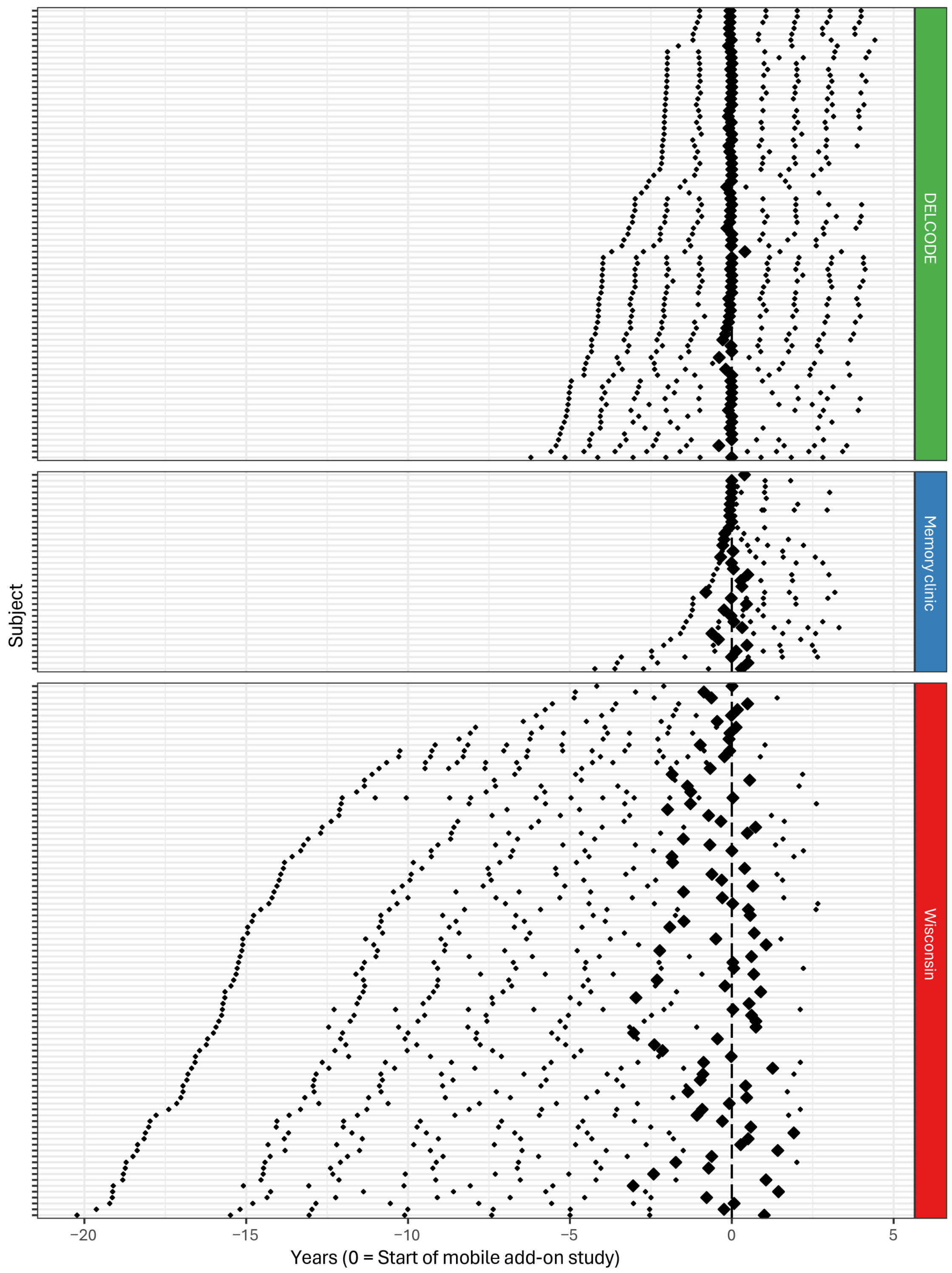

Supplementary Fig. 2 | In-person assessments per subject. Each black diamond indicates an in-person assessment through the parent study or at the memory clinic.

Large black diamonds indicate the chronologically closest in-person appointment to the start of the mobile add-on study. In-person assessments from 4.5 years before and 4.5 years after the start of the mobile-add on study (0 on the x-axis) were considered in the current analyses.

Supplementary Table 15 | Information about in-person measurements, including means and standard deviations of in-person neuropsychological tests per cohort at the in-person appointment chronologically closest to the start of the mobile add-on study, as well as loading obtained in the first step of the lPACC estimation.

	CU	MCI	DELCODE	Memory clinic	Wisconsin
Time points included per person (M ± SD)	4.2 ± 2.3	4.5 ± 1.9	6.6 ± 1.3	3.9 ± 1.4	2.6 ± 1.0
Time range of tests included (years)	−4.5 to 4.2	−4.3 to 4.4	−4.5 to 4.4	−4.2 to 3.3	−4.5 to 2.7
Chronologically closest in-person appointment (years)	−0.24 ± 0.85	−0.05 ± 0.37	−0.05 ± 0.10	0.01 ± 0.30	−0.39 ± 1.09

Task	Obs.	CU	MCI	DELCODE	Memory clinic	Wisconsin	λ
MMSE/MoCA	838	29.5 ± 0.8	26.1 ± 4.0	29.2 ± 1.3	24.5 ± 4.4	29.5 ± 0.8	.901
Fluency: Groceries	532	25.3 ± 5.2	19.0 ± 4.9	24.3 ± 5.5	15.6 ± 5.8	n.a.	.771
Fluency: Phonemic	337	15.5 ± 4.0	9.7 ± 4.5	n.a.	10.1 ± 4.9	15.5 ± 4.0	.704
Fluency: Animals	863	25.0 ± 5.8	17.9 ± 5.7	24.8 ± 6.0	16.7 ± 6.4	23.1 ± 5.5	.637
ADAS-Cog Word Recall	594	8.4 ± 1.6	4.0 ± 2.7	7.7 ± 2.3	3.4 ± 2.7	n.a.	.919
FCSRT96	457	81.8 ± 5.9	67.5 ± 15.0	79.5 ± 8.0	58.2 ± 24.5	n.a.	.882
RAVLT	236	52.0 ± 8.5	29.2 ± 3.7	n.a.	n.a.	51.4 ± 9.2	.819
SDMT	527	51.7 ± 9.7	39.9 ± 12.1	49.7 ± 10.4	32.2 ± 15.8	n.a.	.864
TMT B	769	74.5 ± 30.6	131.5 ± 64.1	91.6 ± 42.7	133.0 ± 66.4	59.9 ± 28.2	−.833
DST	205	55.9 ± 10.4	37.8 ± 9.9	n.a.	n.a.	55.3 ± 10.8	.926
LMDR A/Craft Story 21	233	13.7 ± 3.7	5.5 ± 3.3	n.a.	n.a.	13.5 ± 3.9	.635
LMDR B	534	16.0 ± 4.1	10.2 ± 5.6	15.2 ± 4.5	5.1 ± 5.3	n.a.	.908

Note. MMSE = Mini-Mental State Examination, MoCA = Montreal Cognitive Assessment , ADAS-Cog = Alzheimer's Disease Assessment Scale—Cognitive; FCSRT = Free and Cued Selected Reminding Test, RAVLT = Rey Auditory Verbal Learning Test, SDMT = Symbol Digit Modalities Test, TMT B = Trail Making Test B, DST = Digit Symbol Test, LMDR = Logical Memory Delayed Recall, CU = cognitively unimpaired, MCI = mild cognitive impairment, n.a. = not administered, λ = loading onto the latent Preclinical Alzheimer's Cognitive Composite (lPACC).

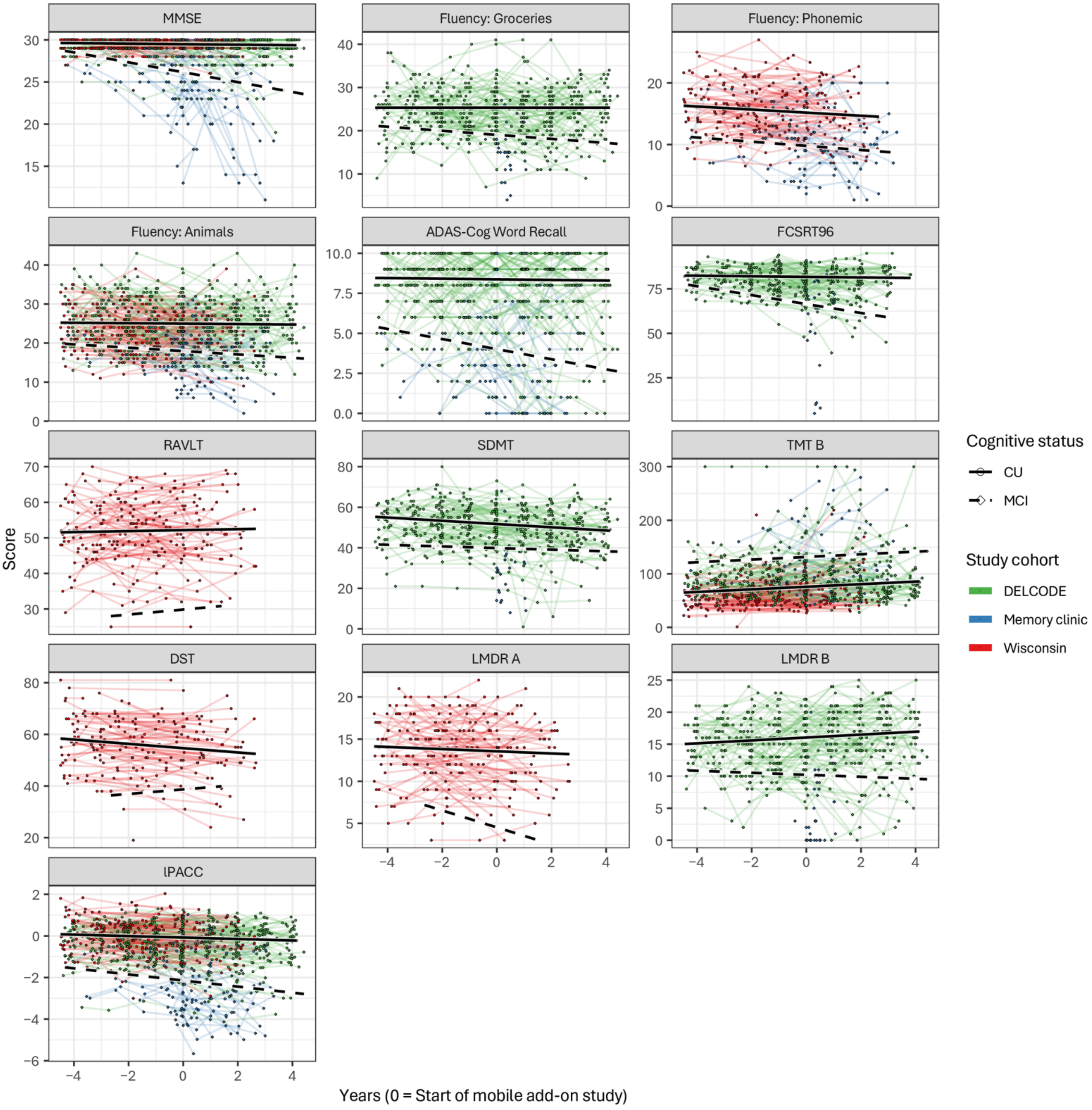

Supplementary Fig. 3 | Raw scores of the in-person neuropsychological assessments as well as the extracted lPACC scores.

MMSE = Mini-Mental State Examination, ADAS-Cog = Alzheimer's Disease Assessment Scale—Cognitive; FCSRT = Free and Cued Selected Reminding Test, RAVLT = Rey Auditory Verbal Learning Test, SDMT = Symbol Digit Modalities Test, TMT B = Trail Making Test B, DST = Digit Symbol Test, LMDR = Logical Memory Delayed Recall, lPACC = latent Preclinical Alzheimer's Cognitive Composite, CU = cognitively unimpaired, MCI = mild cognitive impairment.

Supplementary Table 16 | Number of observations filtered from each task before analyses.

Task	n_{CU}/n_{MCI}	Total obs.	Reason for filtering	Obs. (%) filtered	Obs. (%) included
MDT-OS	137/46	1102	> 17 timeouts during task	1 (< 1%)	1003 (91%)
			Technical error	91 (8%)	
			Only one observation per participant	7 (1%)	
ORR	145/49	1153	Time elapsed before retrieval phase > 96 hours	53 (5%)	1023 (89%)
			Missing retrieval phase	16 (1%)	
			> 8 timeouts during encoding	7 (1%)	
			> 8 timeouts during retrieval	44 (4%)	
			Technical error	2 (< 1%)	
			Only one observation per participant	8 (1%)	
CSR	148/49	1139	Time elapsed before retrieval phase > 96 hours	46 (4%)	1067 (94%)
			Missing retrieval phase	2 (< 1%)	
			> 13 timeouts during encoding	11 (1%)	
			Technical error	2 (< 1%)	
			Only one observation per participant	11 (1%)	

Note. CU = cognitively unimpaired, MCI = mild cognitive impairment, obs. = observations, ORR = Object-in-Room Recall, MDT-OS = Mnemonic Discrimination Task for Objects and Scenes, CSR = Complex Scene Recognition.

Additionally, due to a technical error, one participant completed 18 sessions before being reset and then completed another 24 sessions; only the data from this individual's first 18 sessions are considered.

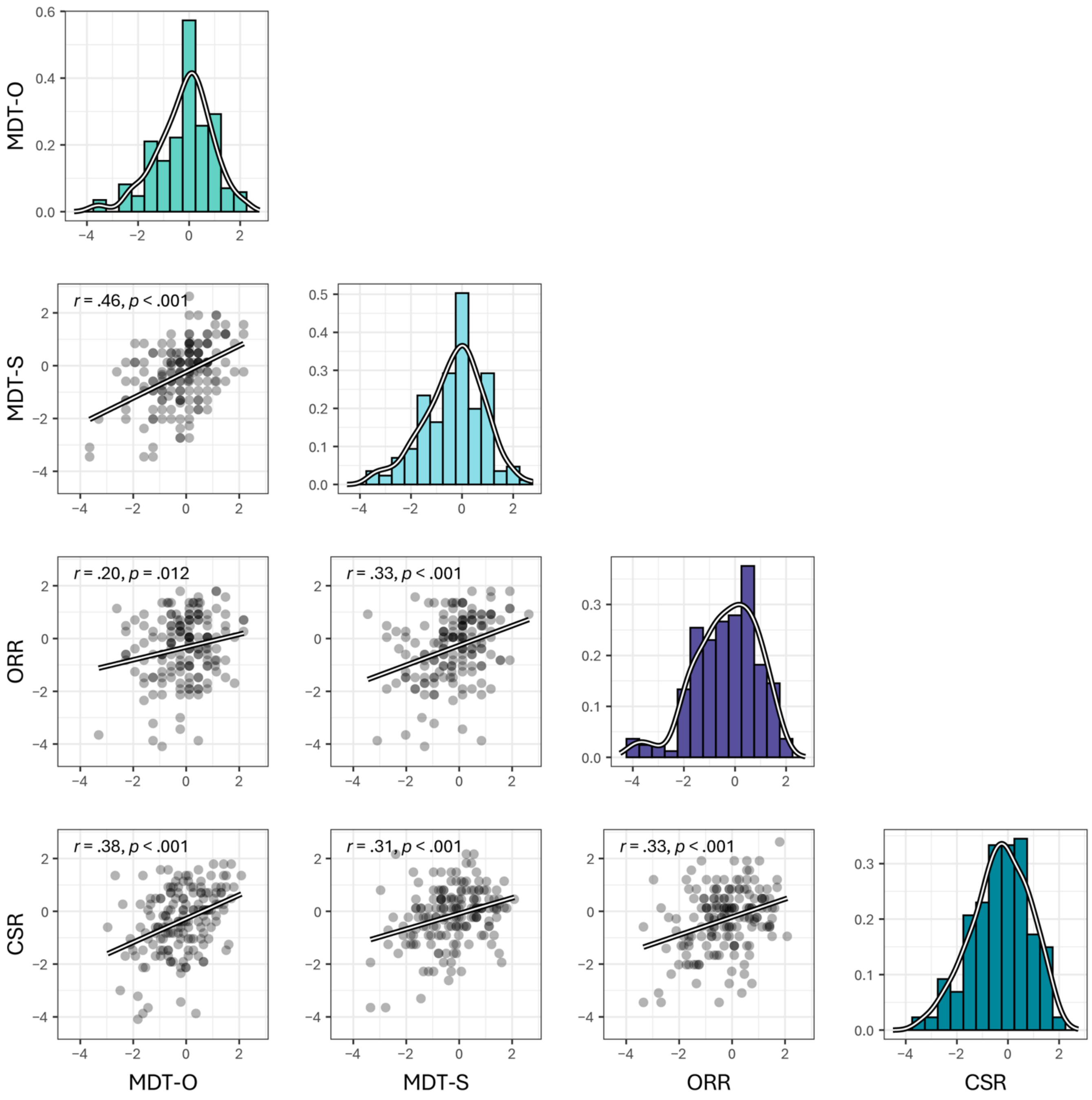

Supplementary Fig. 4 | Pairwise correlations between performance on remote tasks at baseline.

Density plots showing the distribution of task performance (centered to the CU group and scaled) is shown on the diagonal. Pairwise scatterplots are shown in the lower triangle.
MDT-O/-S = Mnemonic Discrimination Task for Objects/Scenes, ORR = Object-in-Room Recall, CSR = Complex Scene Recognition.

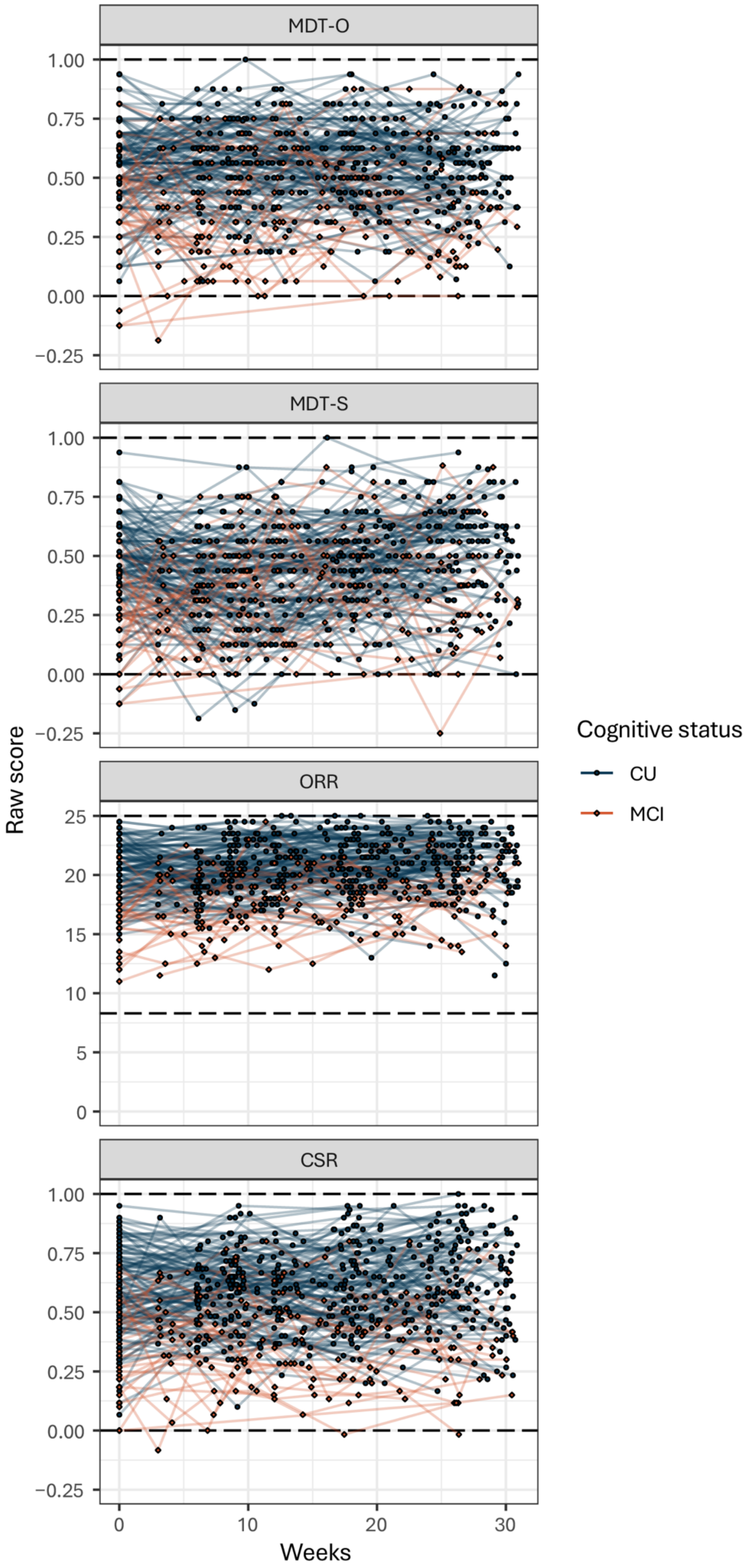

Supplementary Fig. 5 | Raw scores on remote tasks over time.

Spaghetti plots showing individual performance over time on the remote tasks. For the MDT-O/-S, as well as for the CSR, corrected hit rates (hit rate − false alarm rate) are shown. For the ORR, average score between immediate and delayed recall is shown. Higher scores indicate better performance on all tasks. Dashed lines represent chance performance (i.e., floor) and the highest score possible (i.e., ceiling). MDT-O/-S = Mnemonic Discrimination Task for Objects/Scenes, ORR = Object-in-Room Recall, CSR = Complex Scene Recognition.

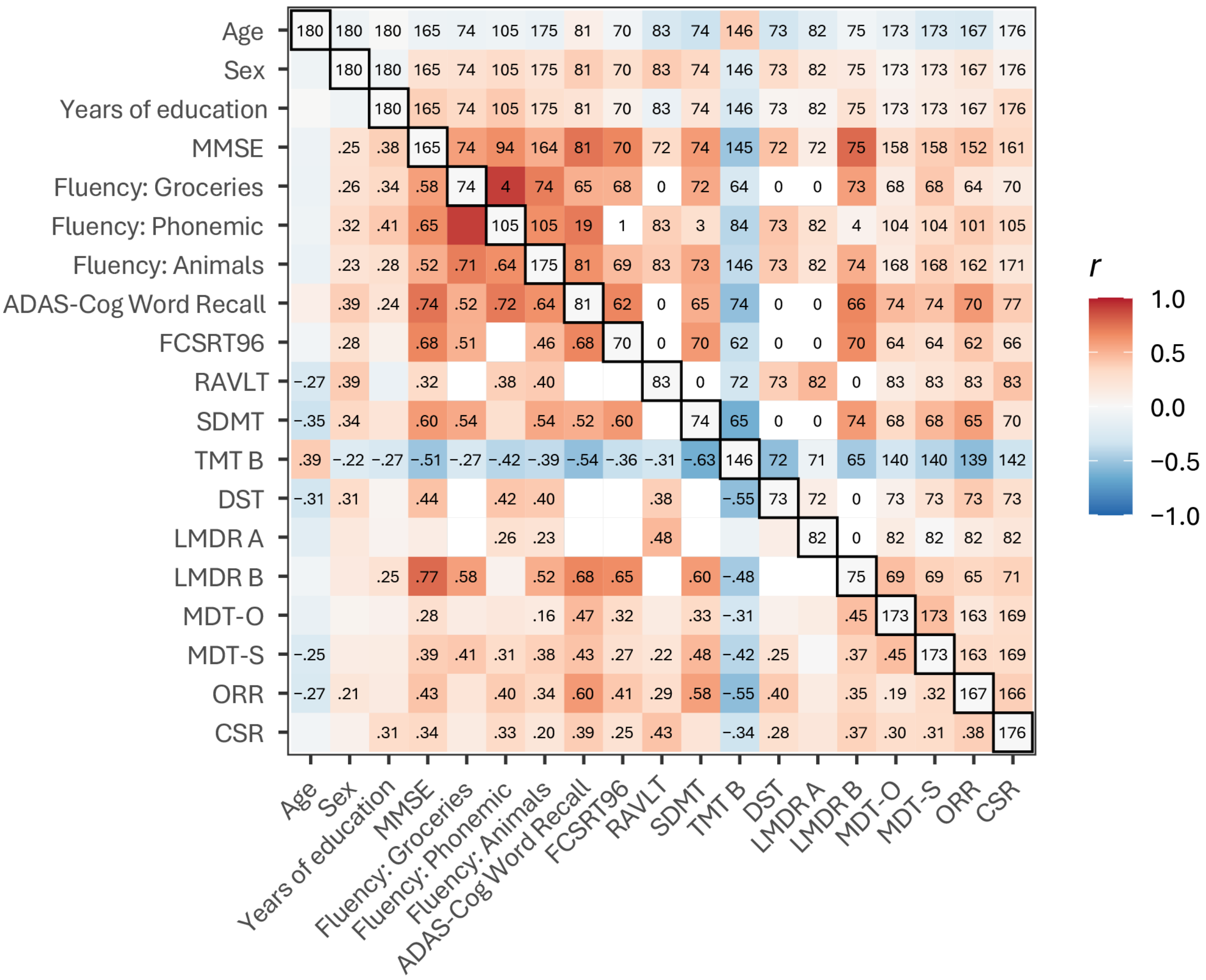

Supplementary Fig. 6 | Pairwise correlations between demographic factors, neuropsychological test scores at the in-person visit chronologically closest to the start of the mobile add-on study, and remote task scores at baseline.

Correlation strength is indicated by color, with red indicating a positive correlation and blue indicating a negative correlation. The *N* of participants included in each pair is given on the diagonal. The *n*s included in each pairwise correlation are found in the upper triangle. Correlation coefficients r of significant pair-wise correlations at $p < .050$ uncorrected are found in lower triangle. Note that some pairs of tests have no overlap (white cells) as they were not administered in the same cohorts. Sex is coded as 0 for male and 1 for female.

MMSE = Mini-Mental State Examination, ADAS-Cog = Alzheimer's Disease Assessment Scale—Cognitive; FCSRT = Free and Cued Selected Reminding Test, RAVLT = Rey Auditory Verbal Learning Test, SDMT = Symbol Digit Modalities Test, TMT B = Trail Making Test B, DST = Digit Symbol Test, LMDR = Logical Memory Delayed Recall, ORR = Object-in-Room Recall, MDT-OS = Mnemonic Discrimination Task for Objects and Scenes, CSR = Complex Scene Recognition.
